# A highly biocompatible polyethyleneimine/sulfonated polysulfone hemoperfusion microsphere with tailored surface charge for rapid and efficient removal of major protein-bound uremic toxins from simulated human plasma

**DOI:** 10.1093/rb/rbaf082

**Published:** 2025-08-08

**Authors:** Shujing Wang, Jiahao Liang, Yu Chen, Xianda Liu, Dongmei Tong, Yupei Li, Weifeng Zhao, Baihai Su, Changsheng Zhao

**Affiliations:** Department of Nephrology, Kidney Research Institute, Frontiers Science Center for Disease-Related Molecular Network, West China Hospital of Sichuan University, Chengdu 610041, China; State Key Laboratory of Advanced Polymer Materials, College of Polymer Science and Engineering, Sichuan University, Chengdu 610065, China; State Key Laboratory of Advanced Polymer Materials, College of Polymer Science and Engineering, Sichuan University, Chengdu 610065, China; State Key Laboratory of Advanced Polymer Materials, College of Polymer Science and Engineering, Sichuan University, Chengdu 610065, China; State Key Laboratory of Advanced Polymer Materials, College of Polymer Science and Engineering, Sichuan University, Chengdu 610065, China; Department of Nephrology, Kidney Research Institute, West China Hospital of Sichuan University, Chengdu 610041, China; State Key Laboratory of Advanced Polymer Materials, College of Polymer Science and Engineering, Sichuan University, Chengdu 610065, China; Department of Nephrology, Kidney Research Institute, Frontiers Science Center for Disease-Related Molecular Network, West China Hospital of Sichuan University, Chengdu 610041, China; Department of Nephrology, Kidney Research Institute, West China Hospital of Sichuan University, Chengdu 610041, China; Med+ Biomaterial Institute of West China Hospital/West China School of Medicine, Sichuan University, Chengdu 610041, China; Med-X Center for Materials, Sichuan University, Chengdu 610041, China; State Key Laboratory of Advanced Polymer Materials, College of Polymer Science and Engineering, Sichuan University, Chengdu 610065, China

**Keywords:** protein-bound uremic toxins, hemoperfusion, adsorption, electrostatic interactions, hemocompatibility

## Abstract

Conventional hemodialysis and hemodiafiltration prove less effective at removing protein-bound uremic toxins (PBUTs) from the bloodstream of end-stage renal disease patients, primarily because PBUTs cannot pass through the small pores in the polymeric membranes. Hemoperfusion is an extracorporeal blood purification technique that employs an adsorption mechanism to remove multiple uremic toxins from such patients. Yet, the efficacy of hemoperfusion is constrained by some limitations of contemporary adsorbents, such as suboptimal capacity to adsorb PBUTs and poor hemocompatibility, presenting significant barriers for their clinical application. To address these challenges, we engineered a tailored hemoperfusion adsorbent by compounding sulfonated polysulfone (SPSf) and polyethyleneimine (PEI) into polyethersulfone (PES) microspheres to effectively capture and remove PBUTs through electrostatic interactions. Specifically, we introduced sulfuric acid into the coagulation bath to increase the adsorption amount of the developed adsorbent (H-PES/SPSf@PEI microspheres), to neutralize strong positive charge of PEI and to improve hemocompatibility. The tailored H-PES/SPSf@PEI microspheres neither damage blood cells nor activate the complement pathway when they contact with human blood. Moreover, H-PES/SPSf@PEI microspheres have a high adsorption amount toward major PBUTs, including hippuric acid (HA, 34.24 mg/g), 3-indoleacetic acid (IAA, 49.19 mg/g), p-cresol sulfate (PCS, 40.31 mg/g) and indoxyl sulfate (IS, 128.67 mg/g) by fitting adsorption isotherms. In a simulated hemoperfusion setting, the removal ratio of IS, IAA, PCS and HA by H-PES/SPSf@PEI microspheres reaches nearly 75.33%, 41.73%, 44.36% and 21.11%, respectively, with 47.89% of IS, 40.64% of IAA, 44.42% of PCS and 37.35% of HA being removed from BSA solution. In conclusion, H-PES/SPSf@PEI microspheres hold a potential to eliminate PBUTs from patients with end-stage renal disease.

## Graphical abstract

**Figure:**
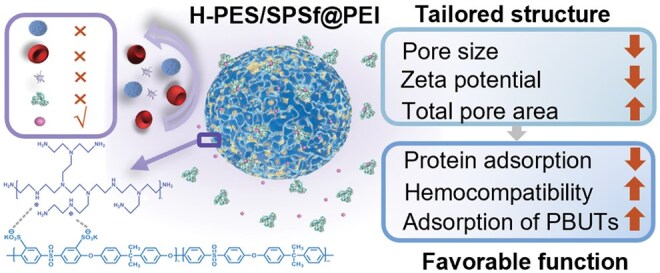

## Introduction

In recent years, chronic kidney disease (CKD) has become a global health problem, and ∼850 million patients suffer from CKD worldwide [1]. When advanced CKD progresses into end-stage renal disease (ESRD) [2], a variety of uremic toxins accumulate in the body to induce a state of chronic inflammation [3], kidney fibrosis [4], and to increase the incidence of cardiovascular diseases and mortality [5]. According to their molecular weight (*M*_W_) and physicochemical characteristics, uremic toxins can be classified into: (i) small water-soluble molecules [*M*_W_<0.5 kDa, i.e. urea, creatinine, uric acid, asymmetric dimethylarginine, trimethylamine N-oxide, etc]; (ii) small-middle molecules [0.5 kDa< *M*_W_ <15 kDa, i.e. β_2_-microglobulin, interleukin-8]; (iii) medium-middle molecules [15 kDa< *M*_W_ <25 kDa, i.e. tumor necrosis factor, interleukin-18, interleukin-6, kappa-free light chain, myoglobin, complement factor D, fibroblast growth factor-2, etc]; (iv) large-middle molecules [25 kDa< *M*_W_ <58 kDa, i.e. pentatraxin-3, fibroblast growth factor-23, lambda-free light chain, chitinase-3-like protein 1, advanced glycosylation end products, interleukin-2, etc]; (v) large molecules [*M*_W_ >58 kDa, modified albumin]; and (vi) small protein-bound uremic toxins (PBUTs) [*M*_W_ <0.5 kDa, i.e. indoxyl sulfate (IS), hippuric acid (HA) p-cresol sulfate (PCS), 3-indoleacetic acid (IAA), homocysteine] [6]. Among them, PBUTs, including IS, PCS, HA, IAA, are a class of small molecule toxins, and most of them possess an aromatic ring structure and anionic functional group. PBUTs have a strong affinity to albumin by binding to Sudlow’s site I (subdomain IIA) and Sudlow’s site II (subdomain IIIA) on albumin through electrostatic interactions, hydrophobic effect and hydrogen bonds [7]. Recently, clinical evidence suggested that the accumulation of PBUTs in ESRD patients can cause chronic ischemic heart disease as well as oxidative stress, and is thus associated with increased cardiovascular mortality in this patient population [8].

Renal replacement therapy, including hemodialysis, peritoneal dialysis and kidney transplantation, has become an effective treatment for ESRD [9]. Although kidney transplantation significantly improves life expectancy and quality of life [10], a chronic and burgeoning shortage of transplantable organs and the need for chronic immunosuppression following transplantation limit its wide use in clinical practice [11]. Peritoneal dialysis has the advantage of being a home-based therapy [12]. However, peritoneal dialysis-related peritonitis is a major concern for ESRD patients, which may result in the cessation of therapy [13]. In contrast, hemodialysis and hemodiafiltration, as the most prevalent renal replacement therapy modalities, can effectively remove small and middle molecule uremic toxins from blood through diffusion and convection, respectively [14]. However, both hemodialysis and hemodiafiltration fail to eliminate PBUTs efficiently because of the strong affinity between PBUTs and plasma proteins [15, 16]. Specifically, contemporary hemodialysis removes only 29% of PCS and 32% of IS, respectively [17], whereas hemodiafiltration is not superior to hemodialysis regarding its efficacy on the removal of these PBUTs [15, 18].

Hemoperfusion utilizes an adsorption mechanism to eliminate harmful solutes through hydrophobic interactions, ionic interactions, *Van der Waals* forces between solutes and solid adsorbents [19]. Compared with hemodialysis and hemodiafiltration, hemoperfusion is more direct, technically relatively simple, and theoretically efficient to remove PBUTs [20]. To date, several adsorbents have been developed to eliminate PBUTs in ESRD patients. Porous materials, such as activated carbons, zeolites and metal organic frameworks (MOFs), are commonly used to fabricate adsorbents for the removal of PBUTs [21–24]. For instance, Raharjo *et al.* [25] incorporated p-cresol-imprinted zeolite and poly(vinyl pyrrolidone) into polyethersulfone (PES) membrane to produce a hollow fiber mixed matrix membrane, which could remove PCS at 186.22 times higher amount than unmodified PES membrane. Ding *et al.* [26] embedded MIL-101(Cr) in polyacrylonitrile to prepare a nanofibrous membrane that shows a high adsorption amount of 103 mg/g for IS. However, porous adsorptive materials mainly bind toxins via *Van der Waals* forces, which significantly lacks adsorption selectivity. Despite MOFs showing better selectivity than activated carbons and zeolites [23, 27], the hemocompatibility, stability and cost of MOFs-based biomaterials remain a great concern.

In addition to porous activated carbons, zeolites and MOFs, polyethyleneimine (PEI), a hydrophilic polycationic molecule [28], is also a common ligand for the binding of PBUTs. PEI-based adsorbents can eliminate negatively charged ions and solutes via electrostatic interactions [29–31]. For instance, Liu *et al.* [32] fabricated an adsorptive flat-sheet membrane by incorporating the complex of PEI, polyphenol and metal ions (Fe^2+^ and Zn^2+^) into the polysulfone membrane to remove PBUTs via π–π and cation–π interaction. In this study, the maximum adsorption amount of the developed flat-sheet membrane for HA, PCS and IS was 78 mg/g, 134 mg/g and 183 mg/g, respectively. Likewise, Shen *et al.* [33] fabricated PEI-modified cationic liposomes, and found that the adsorption reduction rate of such liposomes for IS in dialysate was 57.65 ± 1.74%. However, these PEI-modified liposomes cannot be used as hemoperfusion adsorbents to remove PBUTs directly, as free-form PEI exerts serious cytotoxic effects on living organisms and blood cells [34]. Therefore, it is of great clinical significance to develop a hemocompatible and efficient hemoperfusion adsorbent to eliminate PBUTs from the blood of ESRD patients.

In this work, we fabricated novel PEI-modified PES microspheres (H-PES/SPSf@PEI microspheres) through a non-solvent-induced phase separation and electro-spraying method as potential adsorbents for PBUTs, as PES possesses outstanding chemical, thermal, oxidative hydrolytic stability [35]. Negatively charged sulfonated polysulfone (SPSf) was entirely compatible with PES in the whole range of adding concentration [36], and was thus used to introduce hydrophilic positively charged PEI onto hydrophobic PES microspheres. Sulfuric acid (H_2_SO_4_) was introduced into coagulation bath to improve hemocompatibility of microspheres by neutralizing the strong positive charge of PEI and decreasing the pore size of the H-PES/SPSf@PEI microspheres. The chemical structure, hemocompatibility and PBUTs removal properties of the developed adsorbent are characterized in this study.

## Materials and methods

### Materials

N-methyl pyrrolidone (NMP) and concentrated sulfuric acid were purchased from Chengdu Kelong Chemical Reagent Co., Ltd, (China). PES (Ultrason E6020P) was obtained from BASF Chemical Company (Germany). SPSf (with 30% sulfonated degree) was obtained from Shandong Dechi Technology Company (China). PEI (Mw 10 000, 99%), sodium bicarbonate (NaHCO_3_, AR, ≥99.8%), IAA (98%), HA (98%), PCS (≥98.0%), bovine serum albumin (BSA, ≥98.0%), acetonitrile (99.8%, H_2_O≤0.003%) and sodium dodecyl sulfate (>99%) were acquired from Aladdin Reagent Co., Ltd (China). Phosphate-buffered saline (PBS) and indoxyl sulfonate (IS, 98%) were purchased from Tansoole Platform (China). Reagent kits for fibrinogen, activated partial thromboplastin (APTT), prothrombin time (PT) and thrombin time (TT) were acquired from SIEMENS. Reagent kits for human complement fragment 3a (C3a) and human complement fragment 5a (C5a) were obtained from Thermo Fisher Scientific Co., Ltd, (USA). The lactate dehydrogenase (LDH) regent assay kit was offered by Beyotime Biotechnology Co., Ltd, (China). Deionized water was used throughout the experiments.

### Fabrication of H-PES/SPSf@PEI microspheres

H-PES/SPSf@PEI microspheres were fabricated through a non-solvent-induced phase separation and electrospraying method. First, a blend solution of SPSf, PEI and PES was obtained by dissolving 0.96 g SPSf, 0.48 g PEI, 1.76 g PES pellets into 16.8 g NMP with 24 h of magnetic stirring. Second, the blending solution was dropped into a coagulation bath containing 10 g/L sulfuric acid aqueous solution to obtain H-PES/SPSf@PEI microspheres through electro-spraying at room temperature, with a set voltage of 8.0 kV and a set feeding rate of 1.5 mm/min. Third, these microspheres were further soaked in the coagulation bath for 24 h and then post-treated by with NaHCO_3_ solution to neutralize excessive sulfuric acid. Finally, the obtained H-PES/SPSf@PEI microspheres were washed with DI repeatedly to remove unbound PEI molecules. We further fabricated H-PES, H-PES/SPSf, H-PES/PEI and PES/SPSf@PEI microspheres to investigate the effects of different components of the blend solution on the adsorption behavior of PBUTs and hemocompatibility (see Table 1).

**Table 1.** Formula and specific components of all microspheres

Samples	PES (g)	SPSf (g)	PEI (g)	NMP (g)	Coagulation bath (H_2_SO_4_, g/L)
H-PES	3.20	0	0	16.80	10.00
H-PES/SPSf	2.24	0.96	0	16.80	10.00
H-PES/PEI	2.72	0	0.48	16.80	10.00
PES/SPSf@PEI	1.76	0.96	0.48	16.80	0
H-PES/SPSf@PEI	1.76	0.96	0.48	16.80	10.00

### Characterization of H-PES/SPSf@PEI microspheres

A scanning electron microscope (SEM, Regulus 8220, Japan) was used to observe the surface and cross-sectional morphology of H-PES/SPSf@PEI microspheres. Fourier transform infrared spectroscopy (FTIR, Thermo Scientific Nicolet iS50, ThermoFisher), X-ray photoelectron spectroscopy (XPS, Thermo Fisher K-ALPHA, USA) and thermogravimetric analysis (TGA, METTLER TOLEDO, Switzerland) were used to characterize the chemical structure and thermal properties of H-PES/SPSf@PEI microspheres. Mercury intrusion porosimetry (MIP, AutoPore IV 9500, USA) was employed to confirm the specific surface area, porosity and pore distribution of the microspheres. The compressive strength was characterized by a universal testing machine (HZ-1003-DZ, China). Detailed experimental information for characterization is described in the Supplementary Data.

### Hemocompatibility evaluation assays of H-PES/SPSf@PEI microspheres

All *in vitro* hemocompatibility experiments were performed by West China Hospital, Sichuan University. Collection of fresh blood from healthy volunteers was approved by the Biomedical Research Ethics Committee of West China Hospital of Sichuan University (ethics approval number: No. 2024553) with written consent from donors.

The hemolysis ratio of H-PES/SPSf@PEI microspheres was determined by measuring the absorbance at 540 nm of erythrocyte supernatant after its incubation with the microspheres. In order to measure the surface zeta potential of the microspheres, a surface Zeta Potential analyzer (Surpass 3, Anton Paar) was used. To evaluate the effect of H-PES/SPSf@PEI microspheres on blood cells, we detected the change in the number of leucocyte, erythrocyte and platelet before and after H-PES/SPSf@PEI microspheres contact with EDTA-anticoagulated whole blood via a hematology analyzer (Mindray BC-5100, China). Platelet adhesion on the surface of H-PES/SPSf@PEI microspheres was measured via both an LDH assay kit and SEM observation. The plasma concentrations of C3a and C5a were detected by corresponding ELISA kits to investigate the effect of H-PES/SPSf@PEI microspheres on complement activation. An automated blood coagulation analyzer (CA500, Sysmex, Japan) was utilized to evaluate the anticoagulant properties of H-PES/SPSf@PEI microspheres by detecting APTT, PT, TT and fibrinogen concentration in plasma. The morphology of platelets after H-PES/SPSf@PEI microspheres contacted platelet-rich plasma was observed by SEM. BSA adsorption by H-PES/SPSf@PEI microspheres was evaluated via a BCA^TM^ protein assay kit (Thermo Fisher Pierce). Detailed experimental information for blood collection and hemocompatibility evaluation is described in the Supplementary Data.

### Static adsorption of PBUTs by H-PES/SPSf@PEI microspheres in PBS

To determine the adsorption amount of PBUTs by various microspheres, 50 mg microspheres (wet weight) were incubated with 1 ml PBUTs-spiked aqueous solution (100 mg/L of HA, IAA, PCS and IS, dissolved in PBS) at 37°C for 4 h. The concentrations of PBUTs solution before and after static adsorption were measured by UV spectrophotometry (UV-6100S, Mapada, China) and calculated according to standard curves (see Supplementary Figures S1–S4). The UV absorbance wavelength of HA, IAA, PCS and IS were 228 nm, 280 nm, 210 nm and 278 nm. The removal ratio and adsorption amount of all microspheres for PBUTs can be calculated by the following formulas:


(1) $$\mathrm{Removal}\;\mathrm{ratio}\;(\%)\;=\;\frac{C_{o}-C_{i}}{C_{o}}\times 100\%$$

Where *C_o_* is the initial concentration of PBUTs (mg/L), while *C_i_* represents the final concentration (mg/L).


(2) $$\mathrm{Adsorption}\;\mathrm{amount}\;(\mathrm{mg}/g)\;=\;(C_{o}-C_{i})\times \frac{V}{M}$$

Where *V* represents the volume PBUTs incubated with microspheres (L), *M* is the dry weight of the microspheres (g).

We further investigated the effect of the mass of H-PES/SPSf@PEI microspheres on the adsorption behavior of PBUTs by incubating different dry masses (10 mg, 20 mg, 30 mg, 40 mg) of H-PES/SPSf@PEI microspheres with 1 ml of 100 mg/L PBUTs-spiked PBS solution at 37°C for 4 h. Corresponding removal ratio and adsorption amount for different PBUTs was calculated by formula (1) and (2).

To study the adsorption isotherms of PBUTs by H-PES/SPSf@PEI microspheres, 10 mg H-PES/SPSf@PEI (dry weight) was incubated with 1 ml different concentration (10 mg/L, 20 mg/L, 40 mg/L, 80 mg/L, 160 mg/L and 320 mg/L) PBUTs solution at 37°C for 4 h. The Langmuir and Freundlich isotherm model were defined by following equation:


(3) $$\mathrm{The}\;\mathrm{Freundlich}\;\mathrm{model}:\;\ln q_{e}\;=\;\ln K_{f}\;+\;\ln(C_{e})/n$$

(4) $$\mathrm{The}\;\mathrm{Langmuir}\;\mathrm{model}:\frac{C_{e}}{q_{e}}=\frac{1}{Q_{\max}b}+\frac{C_{e}}{Q_{\max}}$$

Where *q*_e_ represents the adsorption amount of PBUTs to H-PES/SPSf@PEI microspheres (mg/g); *C*_e_ represents the initial concentration of PBUTs (mg/L); *K*_f_ is an empirical constant of Freundlich (mg/g); 1/*n* reflected the surface homogeneity and capture strength; *Q*_max_ is maximum adsorption amount (mg/g) and *b* is the Langmuir constant related to binding energy of the sorption system (L/g).

The Zeta potential of PBUTs solution was measured by dynamic light scattering (Malvern Zetasizer Nano-ZS, UK).To study the adsorption kinetic of PBUTs by H-PES/SPSf@PEI microspheres, 10 mg (dry weight) H-PES/SPSf@PEI microspheres was incubated with 1 ml 300 mg/L PBUTs-spiked PBS at 37°C for different time (5 min, 10 min, 15 min, 30 min, 60 min, 90 min, 120 min, 180 min, 240 min, 360 min and 480 min). The pseudo-first and the pseudo-second-order kinetic models were defined by the following equations:


(5) $$\mathrm{The}\;\mathrm{pseudo}-\mathrm{first}\;\mathrm{kinetic}\;\mathrm{models}:\;\ln(q_{e}-q_{t})\;=\;\ln q_{e}-k_{1}t$$

(6) $$\mathrm{The}\;\mathrm{pseudo}-\mathrm{second}\;\mathrm{kinetic}\;\mathrm{models}:\frac{t}{q_{t}}=\frac{1}{k_{2}{q_{e}}^{2}}+\frac{t}{q_{e}}$$

Where *q*_e_ (mg/g) is the equilibrium adsorption amount and *q_t_* (mg/g) is the amount at the given time *t* (min). The *k*_1_ (h ^−1^) and *k*_2_ (g/mg⋅h) are relevant parameters in the models.

### *In vitro* simulated hemoperfusion experiment for the removal of PBUTs in PBS

To simulate extracorporeal hemoperfusion therapy, an *in vitro* hemoperfusion cartridge containing 600 mg (dry weight) of H-PES/SPSf@PEI microspheres was built to eliminate 60 ml of PBUTs-spiked PBS solution (25 mg/L IS, 25 mg/L PCS, 25 mg/L IAA and 70 mg/L HA) for 4 h [37, 38]. The concentrations of PBUTs solution at different time points (5, 10, 15, 30, 60, 60, 90, 120, 180 and 240 min) were measured. Corresponding removal ratio and adsorption amount for different PBUTs were calculated by formula (1) and (2).

### Competitive adsorption of PBUTs by H-PES/SPSf@PEI microspheres in BSA solution

We investigated the PBUTs adsorption property of H-PES/SPSf@PEI microspheres in BSA solution to further highlight their potential to remove bound PBUTs from the blood of ESRD patients. First, a mixture of BSA (40 g/L) and PBUTs (25 mg/L IS, 25 mg/L PCS, 25 mg/L IAA and 70 mg/L HA) in PBS was incubated at 37°C for 24 h to achieve saturation uptake [32] (Supplementary Figure S5). Then, a designated dry mass of H-PES/SPSf@PEI microspheres (10 mg, 20 mg, 40 mg, 60 mg and 80 mg) were incubated with 1 ml of the obtained BSA-PBUTs solution at 37°C for another 4 h. Subsequently, the BSA-PBUTs solution was separated from H-PES/SPSf@PEI- microspheres, and acetonitrile (with a volume ratio of 1:1) was added to the solution. After 3-min vortex, the supernatant was transferred to an ultrafiltration centrifuge tube (4 ml, 10K, Millipore) [38]. All ultrafiltration centrifuge tubes were then centrifuged at 3500 ×g for 15 min. The absorbance of the centrifuged solution was measured by UV spectrophotometry and the removal ratio of IS by H-PES/SPSf@PEI microspheres was calculated.

### Statistical analysis

Data are expressed as mean±SD, indicated by error bars in all graphs. One-way analysis of variance was used to assess the statistically significant differences between multiple groups, while unpaired *t*-test was performed to compare differences between two experimental groups. GraphPad Prism was used for statistical analysis. Statistical significance is indicated as follows: * for *P* < 0.05, ** for *P *< 0.01, *** for *P *< 0.001, **** for *P *< 0.0001 and ns for no significant difference when *P *> 0.05.

## Results and discussion

### Preparation and characterization of H-PES/SPSf@PEI microspheres

**Figure 1.**
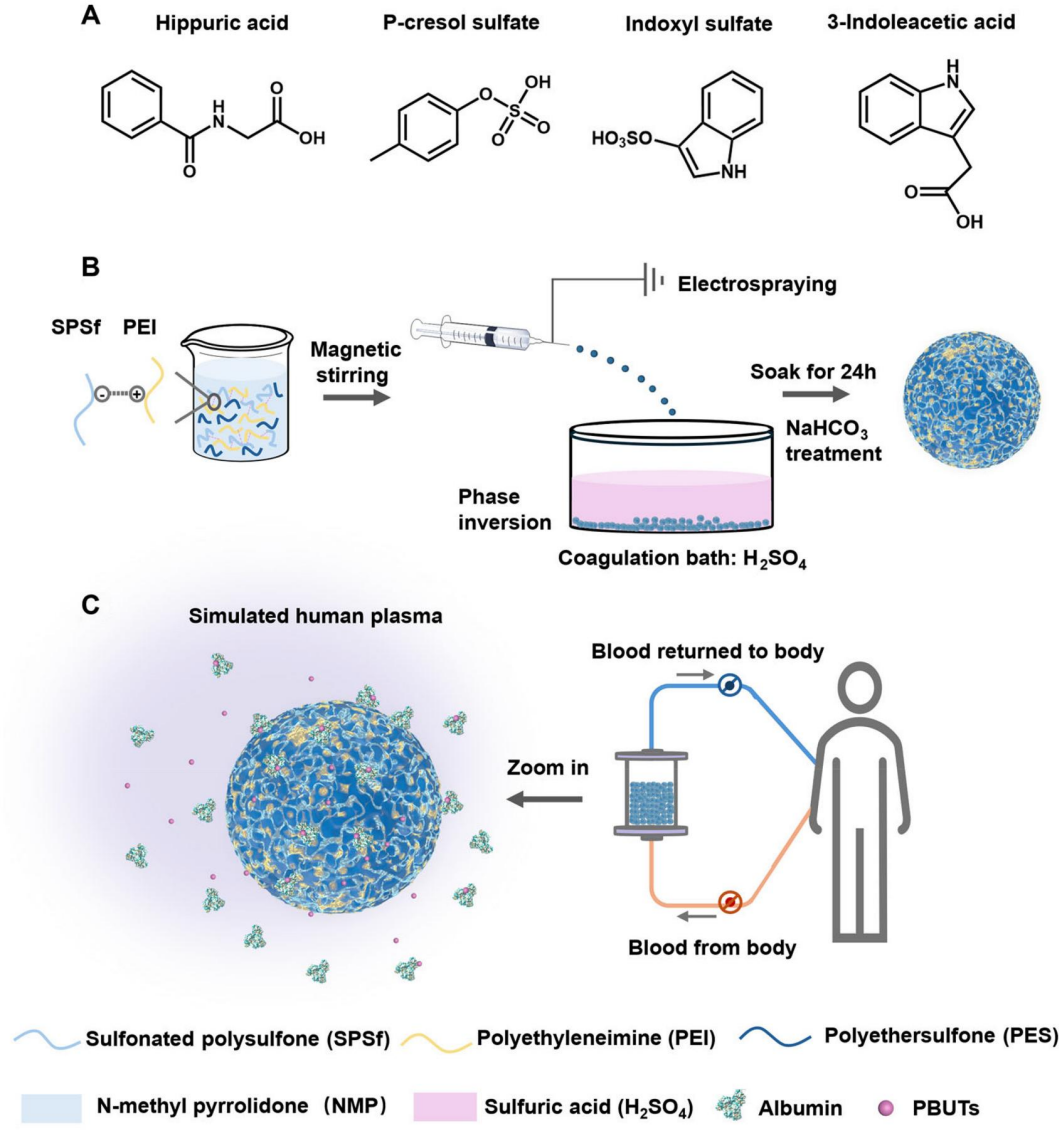
Design of H-PES/SPSf@PEI microspheres for the removal of PBUTs from blood. (**A**) Chemical structures of HA, PCS, IS and IAA. (**B**) Schematic illustration of the fabrication of H-PES/SPSf@PEI microspheres. (**C**) Adsorption of PBUTs from simulated human plasma.

In this study, four representative PBUTs—IS, PCS, IAA and HA—were selected for adsorption studies (Figure 1A). The H-PES/SPSf@PEI microspheres were prepared using an electrospraying-assisted non-solvent-induced phase separation method (Figure 1B). Positively charged PEI, as an adsorption ligand for PBUTs, was immobilized into PES microspheres by negatively charged SPSf through electrostatic interactions. Specifically, a 10-g/L sulfuric acid aqueous solution was employed as the coagulation bath to improve the hemocompatibility and reduce protein adsorption of H-PES/SPSf@PEI microspheres by neutralizing the strong positive charge of PEI and decreasing the pore size of the H-PES/SPSf@PEI microspheres. We anticipated the positively charged PEI molecules on the surface of H-PES/SPSf@PEI microspheres could significantly adsorb PBUTs from the blood of ESRD patients, without affecting other blood components, such as plasma albumin, complement proteins and blood cells (Figure 1C).

**Figure 2.**
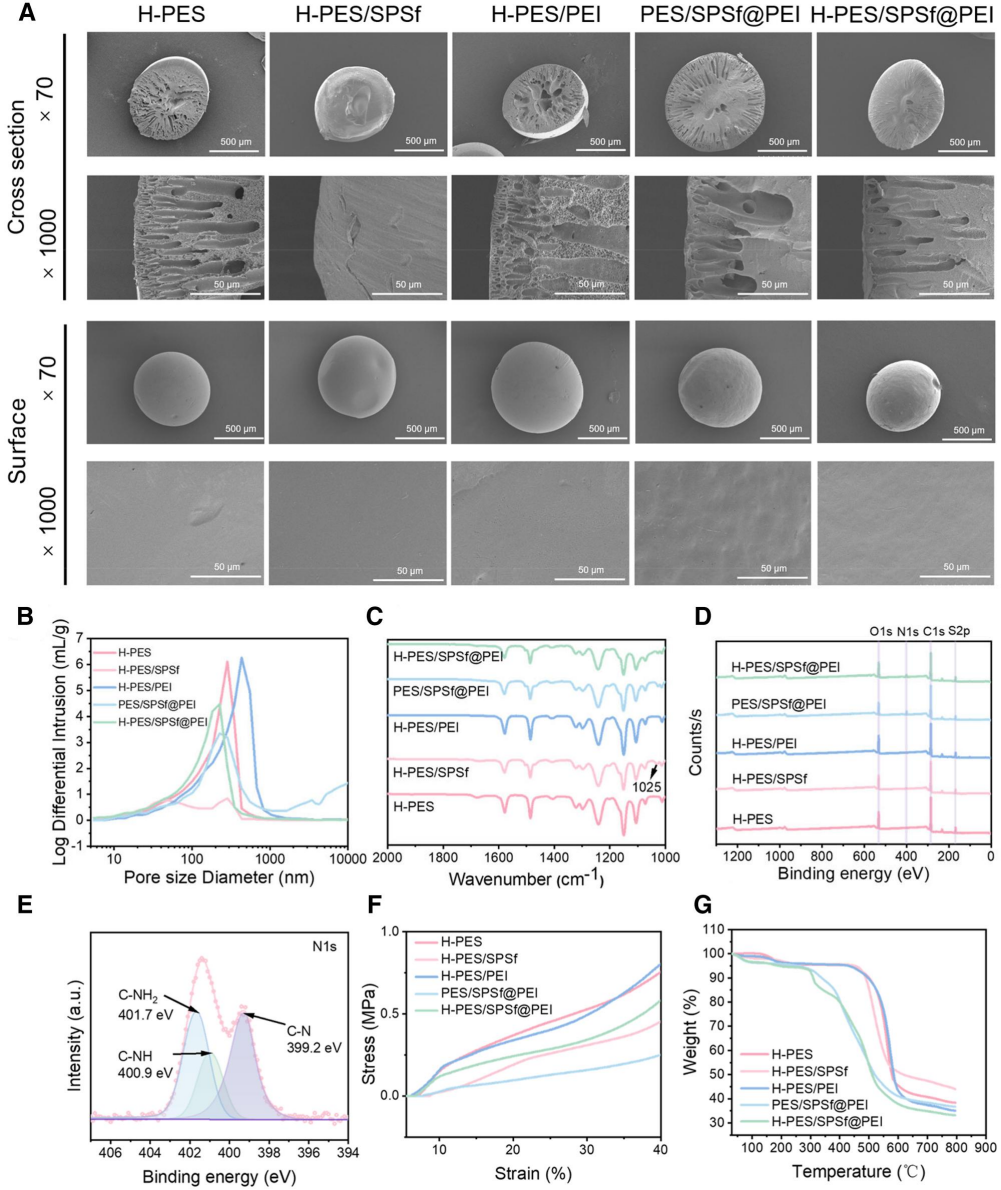
Characterization of all microspheres. (**A**) Surface and cross-sectional SEM images of all microspheres. (**B**) Pore size distribution curves of all microspheres. (**C**) FTIR spectra of all microspheres in the range of 2000–1000 cm^−1^. (**D**) XPS survey spectra of all microspheres. (**E**) The high-resolution XPS spectra of N1s of H-PES/SPSf@PEI microspheres. (**F**) Strain–stress curves of all microspheres. (**G**) TGA curves of all microspheres.

The particle size distributions of the H-PES/SPSf@PEI microspheres are uniform (Supplementary Figure S6). As shown in Figure 2A, all microspheres had a dense texture. The good compatibility and strong interaction force between PES and SPSf could lead to an increase in the viscosity of the PES/SPSf blend solution, which delayed the rapid intrusion of non-solvent and the mutual diffusion of solvent and non-solvent during the phase separation [39]. Therefore, the pore structure of PES microspheres disappeared with the addition of SPSf into PES solution. However, when water-soluble PEI was added into the PES/SPSf blend solution, finger-like pores reappeared. This phenomenon might be due to a better compatibility between PEI and non-solvents [40]. With the sulfuric acid was added in coagulation bath, it could be observed that the finger-like pores of the microspheres became narrower due to the increased diffusion rate between solvent and non-solvent during phase separation [41].

The pore size distribution of all microspheres was measured by MIP. As shown in Figure 2B and Supplementary Table S1, H-PES/SPSf@PEI microspheres possessed the largest total pore aera of 124.799 m^2^/g at 32 993.31 psia, which indicated H-PES/SPSf@PEI microspheres had a large contact area when they came into contact with whole blood. It can be noted that the addition of PEI increased the porosity and average pore size of the microspheres, but the addition of the SPSf and sulfuric acid decreased the porosity and average pore size. The pore size distribution patterns of all microspheres are consistent with the results of SEM observation.

FTIR spectra of the microspheres are shown in Figure 2C. Compared with H-PES and H-PES/PEI microspheres, distinct peaks at 1025 cm^−1^ were present in the spectra of H-PES/SPSf, PES/SPSf@PEI and H-PES/SPSf@PEI microspheres, which were attributed to symmetric stretching vibrations of S = O in -SO_3_H [42]. These results indicated the successful introduction of SPSf into H-PES/SPSf, PES/SPSf@PEI and H-PES/SPSf@PEI microspheres.

XPS was employed to further demonstrate the elemental composition and chemical bonding of H-PES/SPSf@PEI microspheres. As depicted in Figure 2D, PES/SPSf@PEI and H-PES/SPSf@PEI microspheres exhibited a distinct N1s peak, indicative of the presence of nitrogen, suggesting the successful introduction of PEI into both microspheres. Conversely, H-PES, H-PES/SPSf and H-PES/PEI microspheres lacked this characteristic peak. Figure 2E further shows the N1s high-resolution XPS spectrum specific to H-PES/SPSF@PEI microspheres. Here, peaks positioned at 401.7 eV, 400.9 eV and 399.2 eV were attributed to -NH_2_, -NH- and >N-, respectively [43]. Table 2 summarizes the element contents of C, N, O and S in all microspheres. The percentage of N elements in PES/SPSF@PEI and H-PES/SPSF@PEI microspheres was 5.77% and 4.69%, respectively. Notably, nitrogen was not detected in the XPS full spectra of H-PES/PEI microspheres because water-soluble PEI could leak out from PES microspheres during the phase separation and washing process. These findings indicated that SPSf was essential to introduce PEI into PES/SPSf@PEI and H-PES/SPSf@PEI microspheres through electrostatic interactions. Furthermore, we carried out split-peak analysis on the high-resolution XPS spectra of H-PES/SPSf@PEI microspheres for C1s, S2p and O1s (Supplementary Figure S7A–C).

**Table 2.** The percentage of compositions of C, N, O and S elements in microspheres

Sample	C (%)	N (%)	O (%)	S (%)
H-PES	74.6	–	18.92	5.86
H-PES/SPSf	75.15	–	17.92	5.14
H-PES/PEI	73.55	–	20.12	5.73
PES/SPSF@PEI	75.19	5.77	14.97	4.06
H-PES/SPSF@PEI	70.3	4.69	19.64	5.39

Data were derived from XPS analysis.

Subsequently, we evaluated the mechanical strength of all microspheres through a universal testing machine. As shown in Figure 2F, compared to H-PES and H-PES/PEI microspheres, H-PES/SPSf, PES/SPSf@PEI and H-PES/SPSf@PEI microspheres exhibited a notable decline in mechanical strength with the introduction of SPSf into PES matrix. Interestingly, compared with PES/SPSf@PEI microspheres, H-PES/SPSf@PEI microspheres exhibited a considerable increase in mechanical strength upon the addition of sulfuric acid to the coagulation bath. This phenomenon might be attributed to a denser pore structure of H-PES/SPSf@PEI microspheres that enhanced their mechanical properties. It should be noted that H-PES/SPSf@PEI microspheres demonstrated an impressive resistance threshold of 66.7 KPa, an ultimate pressure experienced during a hemoperfusion process.

The TGA and its derivative technique (DTG) curve of all microspheres are shown in Figure 2G and Supplementary Figure S7D. Notably, the TGA curve of H-PES, H-PES/SPSf and H-PES/PEI microspheres manifested a slight reduction in mass within the temperature range of 100–200°C, which could be attributed to the evaporation of residual solvent (NMP) and water molecules. In H-PES and H-PES/PEI microspheres, the second stage of mass loss was around 550°C due to the thermal decomposition of PES [44]. In contrast, the second stage mass loss of H-PES/SPSf microspheres occurred at 520°C, suggesting that the integration of SPSf into PES matrix significantly decreased the thermal stability of H-PES microspheres. The microspheres containing PEI had five important loss stages. The first stage occurred at around 100°C, which was mainly was due to the evaporation of adsorbed water. The second stage at about 100–200°C is because the loss of amine group [40] and solvent, and the third, fourth and fifth steps were attributed to the degradation of PEI, SPSf and PES.

### Hemocompatibility of H-PES/SPSf@PEI microspheres

Hemocompatibility stands as a cornerstone attribute for biomaterials intended for interaction with blood. Hemoincompatible biomaterials can cause platelet activation, protein adsorption and activation of the immune and coagulation system to induce oxidative stress, chronic inflammation and thrombus [45, 46]. Therefore, to verify the safety profiles of H-PES/SPSf@PEI microspheres, we performed comprehensive experiments to evaluate their hemocompatibility according to previous reports [47–50].

**Figure 3.**
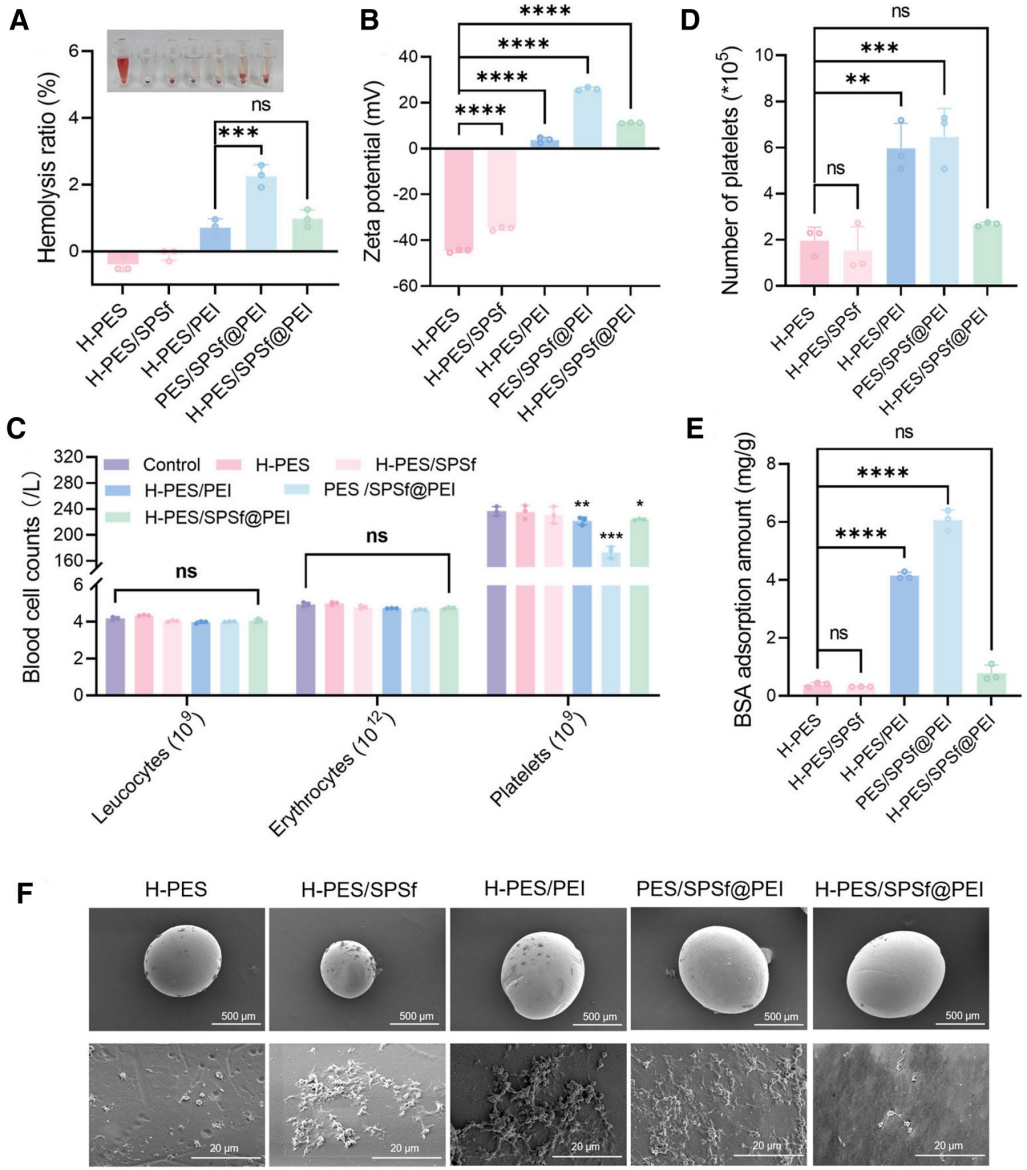
Effect of microspheres on blood cell counts, protein adsorption and platelet adhesion. (**A**) Hemolysis ratio of all microspheres (*n* = 3). (**B**) Zeta potential of all microspheres (*n* = 3). (**C**) Number of leucocytes, erythrocytes and platelets for all microspheres in whole blood (*n* = 3). (**D**) Number of platelets adhering to the microspheres (*n* = 3). (**E**) BSA adsorption amount of all microspheres (*n* = 3). (**F**) SEM images of platelets adhering to the microspheres. * For *P* < 0.05, ** for *P *< 0.01, *** for *P *< 0.001, **** for *P *< 0.0001 and ns for no significant difference when *P *> 0.05.

Excessive hemolysis induced by hemoincompatible adsorbents, if unmitigated, could cause impaired oxygen transport, release of free hemoglobin and subsequent organ dysfunction and systemic reactions. Therefore, we first determined the hemolysis ratio of different microspheres. As shown in Figure 3A, hemolysis ratios of all microspheres were under the threshold of 5%, which fulfilled the standard outlined by the American Society for Testing and Materials (ASTM F756-2008), a widely recognized benchmark in biomaterial–blood interaction standards. Notably, the erythrocyte cell membrane is negatively charged due to the abundant presence of neuraminic acids. Cationic PEI that bears a high surface charge density can thus generate a formidable attraction with erythrocytes to cause cell membrane disruption and hemolysis [51]. Accordingly, among all microspheres under investigation, PES/SPSf@PEI microspheres exhibited a relatively elevated hemolysis ratio (2.26%) due to a pronounced positive charge on their surfaces (Figure 3B). In contrast, the tailored H-PES/SPSf@PEI microspheres, with reduced surface charge, denoted a minimal propensity to cause hemolysis. Furthermore, the negative hemolysis rates of H-PES and H-PES/SPSf microspheres may be attributed to two concurrent mechanisms: (i) electrostatic repulsion between the negatively charged microspheres and the negatively charged erythrocyte membranes, which mitigates membrane damage and cellular lysis [52, 53]; (ii) non-selective hemoglobin adsorption by porous structures of both microspheres [54, 55]. These combined effects result in a lower hemoglobin absorbance of the test samples compared to the negative control group, thereby yielding negative hemolysis rate values.

We further investigated the impact of all microspheres on blood cell counts by determining the change in the number of erythrocytes, leukocytes and platelets after the incubation of whole blood with various microspheres, as shown in Figure 3C. For H-PES/SPSf@PEI microspheres, the numbers of erythrocytes and leukocytes were not significantly decreased despite H-PES/SPSf@PEI microspheres slightly reduced platelet counts compared with the control group. In contrast, both H-PES/PEI and PES/SPSf@PEI microspheres led to a significant reduction in platelet counts, which was in line with the results of our platelet adhesion and BSA adsorption assay that both microspheres markedly adhered more platelets (Figure 3D) and BSA (Figure 3E) than H-PES, H-PES/SPSf and H-PES/SPSf@PEI microspheres. To be specific, for H-PES/PEI (3.70 mV), PES/SPSf@PEI (26.01 mV) and H-PES/SPSf@PEI (11.24 mV) microspheres, the presence of cationic PEI molecules on the surface of H-PES (−44.60 mV) or H-PES/SPSf (−34.90 mV) microspheres was associated with a significant increase in Zeta potential. With an addition of H_2_SO_4_ in the coagulation bath, Zeta potential and average pore size (see Supplementary Table S1) of H-PES/SPSf@PEI microspheres were significantly lower than those of PES/SPSf@PEI microspheres. Therefore, both H-PES/PEI and PES/SPSf@PEI microspheres caused more profound platelet adhesion and BSA adsorption than H-PES/SPSf@PEI microspheres. These findings are consistent with earlier reports that positively charged surfaces and larger pore size of biomaterials are more likely to adsorb negatively charged platelets and BSA [45, 56].

Platelet SEM images were also employed to study the platelet adhesion and activation behavior on the surface of H-PES/SPSf@PEI microspheres. As shown in Figure 3F, SEM images revealed a pronounced platelet adhesion and activation on H-PES/SPSf, H-PES/PEI and PES/SPSf@PEI microspheres, as evidenced by an increased number in pseudopodia and adhered platelets. In comparison, both H-PES and H-PES/SPSf@PEI microspheres showed insignificant platelet adhesion and activation. Altogether, our results showed that H-PES/SPSf@PEI microspheres had a favorable performance in mitigating platelet adhesion and protein fouling.

**Figure 4.**
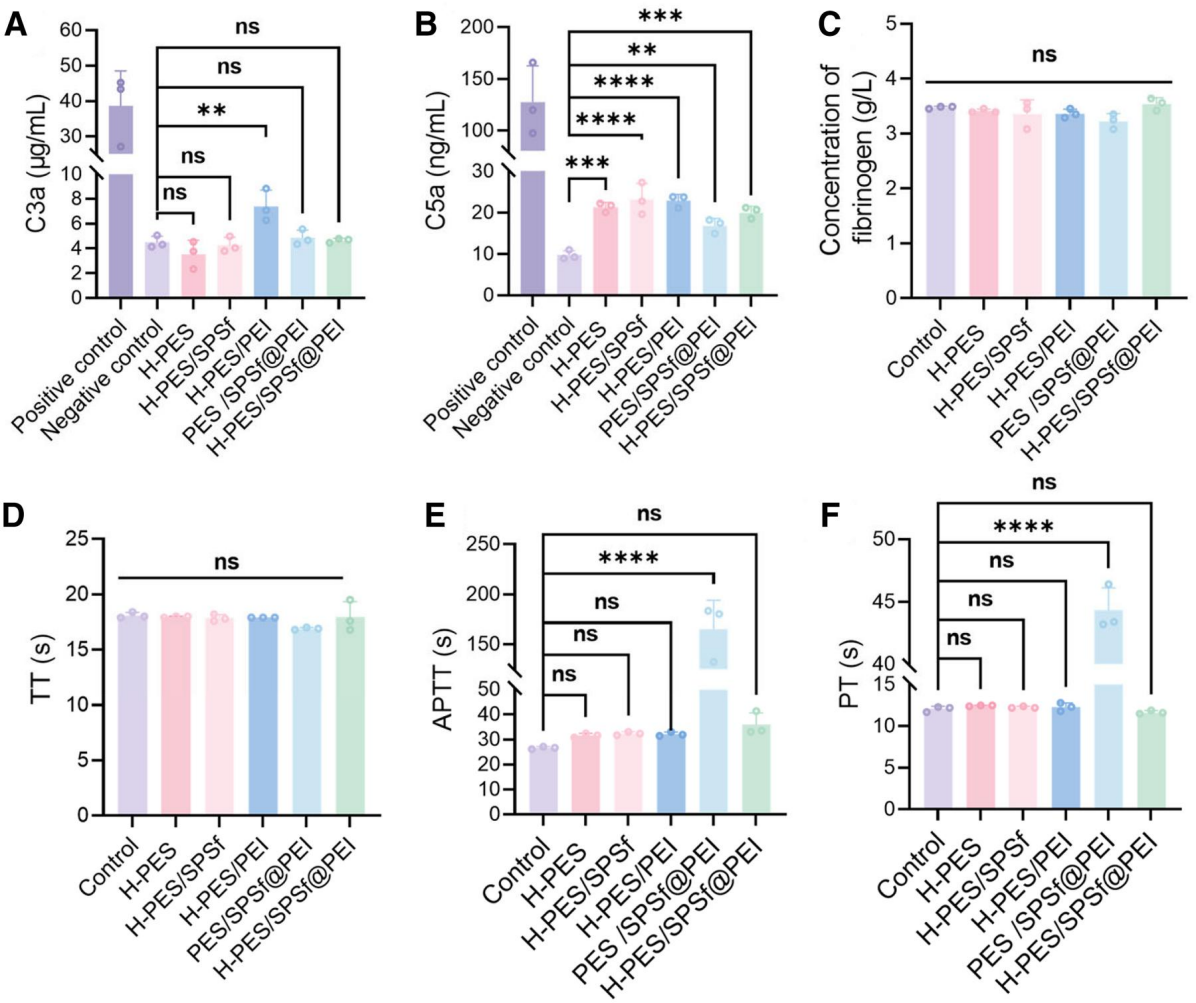
Effect of microspheres on complement activation and anticoagulant activity. Generated concentration of C3a (**A**) and C5a (**B**) after incubating the microspheres with whole blood (*n* = 3). (**C**) The concentration of fibrinogen. The TT values (**D**), APTT values (**E**) and PT values (**F**) (*n* = 3). ** For *P *< 0.01, *** for *P *< 0.001, **** for *P *< 0.0001 and ns for no significant difference when *P *> 0.05.

The complement system plays a vital role in our innate defense. However, biomaterials can cause excessive activation of complement proteins that further contribute to hyperinflammation and tissue damage [57]. In this work, both C3a and C5a, known for their potent effect in chemotaxis and immune cell activation, were used to evaluate the complement activation. Normally, whole blood was employed as the negative control, while cobra venom factor served as the positive control. As shown in Figure 4A, H-PES/SPSf@PEI microspheres did not cause a significant increase in plasma C3a concentration compared to the negative control group. This finding suggests that these microspheres do not appreciably activate the early stage of the complement cascade. However, the concentration of C5a was observed to increase in the H-PES/SPSf@PEI group (Figure 4B). Notably, H-PES/SPSf@PEI microspheres showed no significant difference in the concentrations of either C3a or C5a when compared to H-PES microspheres. These results underscored that H-PES/SPSf@PEI microspheres had a minimal activation of both the early (C3a) and late (C5a) stages of the complement cascade.

In the end, we comprehensively evaluated the anticoagulant activity of all microspheres by measuring APTT, PT, TT and fibrinogen concentration in blood samples. APTT and PT are used to evaluate the intrinsic and extrinsic coagulation pathway, respectively, while TT reflects the time when fibrinogen is converted to fibrin [58]. As illustrated in Figure 4C and D, the plasma fibrinogen levels and TT values of all microspheres were not significantly different from the control group, indicating that these microspheres did not reduce fibrinogen levels nor interfere with the process of fibrin formation. For H-PES, H-PES/SPSf, H-PES/PEI and H-PES/SPSf@PEI microspheres, the APTT and PT values were not significantly different from those of the control. In contrast, after co-incubation with PES/SPSf@PEI microspheres, plasma APTT and PT values were remarkably prolonged from 26.7 to 165.3 s, and from 12.1 to 44.3 s, respectively, compared to the control (Figure 4E and F). This prolongation suggests that PES/SPSf@PEI microspheres have a more pronounced effect on the coagulation pathways. To verify the cause of the prolonged APTT and PT in PES/SPSf@PEI microspheres, the PES/SPSf@PEI microspheres were subjected to post-treatment with a sulfuric acid solution. As shown in Supplementary Figure S8, the APTT and PT values of PES/SPSf@PEI microspheres significantly decreased after sulfuric acid treatment, indicating that excessively strong surface positive charge may affect the coagulation system by inactivating coagulation factors [59, 60]. Altogether, our results showed that H-PES/SPSf@PEI microspheres did not disrupt the coagulation and complement pathways, thereby minimizing the risk of thrombosis and other blood–material interactions.

### *In vitro* static adsorption of PBUTs by H-PES/SPSf@PEI microspheres in PBS

**Figure 5.**
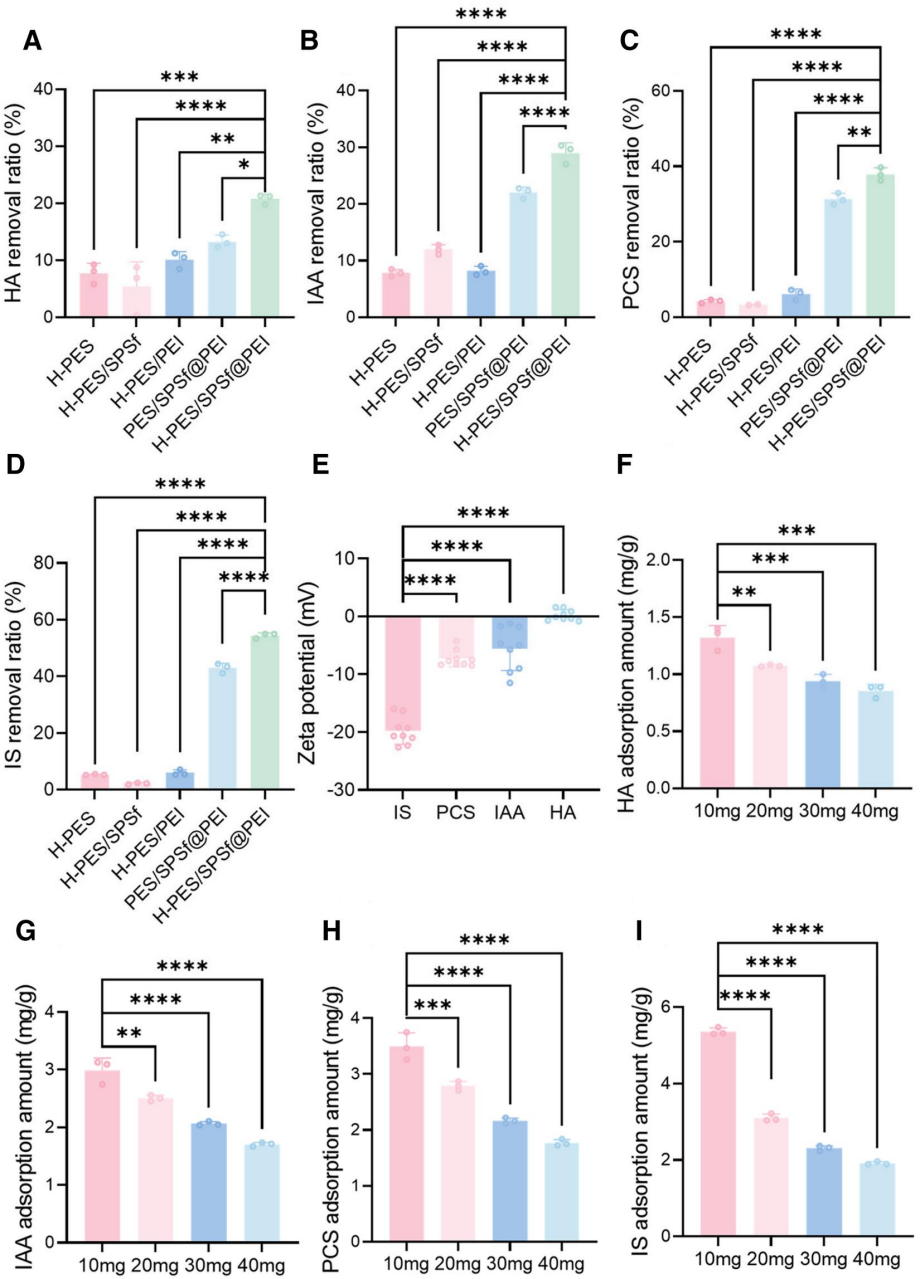
Adsorption of PBUTs by H-PES/SPSf@PEI microspheres in PBS. Removal ratio of HA (**A**), IAA (**B**), PCS (**C**) and IS (**D**) by all microspheres (*n* = 3). (**E**) Zeta potential of 100 mg/L PBUTs in water (*n* = 9). Adsorption amount of HA (**F**), IAA (**G**), PCS (**H**) and IS (**I**) by H-PES/SPSf@PEI microspheres of different dry mass (*n* = 3). * For *P* < 0.05, ** for *P *< 0.01, *** for *P *< 0.001, **** for *P *< 0.0001 and ns for no significant difference when *P *> 0.05.

As most PBUTs are negatively charged under physiological conditions, in this work, we fabricated PEI-immobilized PES-based microspheres to absorb PBUTs via electrostatic interactions. First, we conducted a 4-h static batch adsorption experiment to investigate the impact of various components on the adsorption behavior of PBUTs. As shown in Figure 5A–D, H-PES/SPSf@PEI microspheres (adsorption of 1 ml of 100 mg/L PBUTs solution by 50 mg wet weight microspheres) showed the highest adsorption removal ratio for HA (20.80%), IAA (29.00%), PCS (37.90%) and IS (54.44%) when compared to the other four microspheres. Compared to H-PES/SPSf@PEI microspheres, PES/SPSf@PEI microspheres exhibited higher positive surface charge, larger pore size and greater porosity. However, contrary to expectations, they demonstrated lower adsorption capacities for major PBUTs. This apparent paradox may be explained by the significantly larger total pore area of H-PES/SPSf@PEI microspheres, which appears to be the dominant factor for small molecules (free-form PBUTs) adsorption. In contrast, Figure 3D and E reveal that H-PES/PEI microspheres (+3.70 mV) showed superior adsorption capacities for both negatively charged macromolecular BSA (66.4 kDa) and platelets compared to H-PES/SPSf@PEI microspheres (+11.24 mV). This reversal in adsorption performance can be attributed to their larger pore size and higher porosity, which are critical for accommodating larger blood components such as albumin and platelets. Among the four tested PBUTs, it could be observed that H-PES/SPSf@PEI microspheres performed the highest removal ratio for IS and the worst removal ratio for HA. We further conducted a Zeta potential analysis of four PBUTs in an aqueous solution at a concentration of 100 mg/L. As shown in Figure 5E. IS displayed an apparent negative charge, while HA was nearly electroneutral, suggesting that non-static-electric interaction was needed for HA adsorption clearance. This finding indicated that other factors, such as hydrophobic interactions or hydrogen bonding, also played a crucial role in the adsorption process. Of note, the developed H-PES/SPSf@PEI microspheres showed stable adsorption performance for PBUTs as the removal ratio of IS by H-PES/SPSf@PEI remained consistent over a 1-month storage period (Supplementary Figure S9). Overall, we showed that H-PES/SPSf@PEI microspheres had the highest adsorption removal ratio for major PBUTs in PBS solution among all microspheres.

Then, the effect of adsorbent mass on the removal efficiency of PBUTs was investigated by varying the dosage of H-PES/SPSf@PEI microspheres from 10 to 40 mg. As shown in Figure 5F–I, the adsorption amount of four major PBUTs decreased as the mass of microsphere increased. However, the removal ratio of such PBUTs increased with the increasing mass of H-PES/SPSf@PEI microspheres, suggesting a significant dose-dependent manner (Supplementary Figure S10). Based on these results, subsequent adsorption experiments were performed using 10 mg (dry weight) of H-PES/SPSf@PEI microspheres.

### Adsorption isotherms and kinetics of PBUTs by H-PES/SPSf@PEI microspheres in PBS

**Figure 6.**
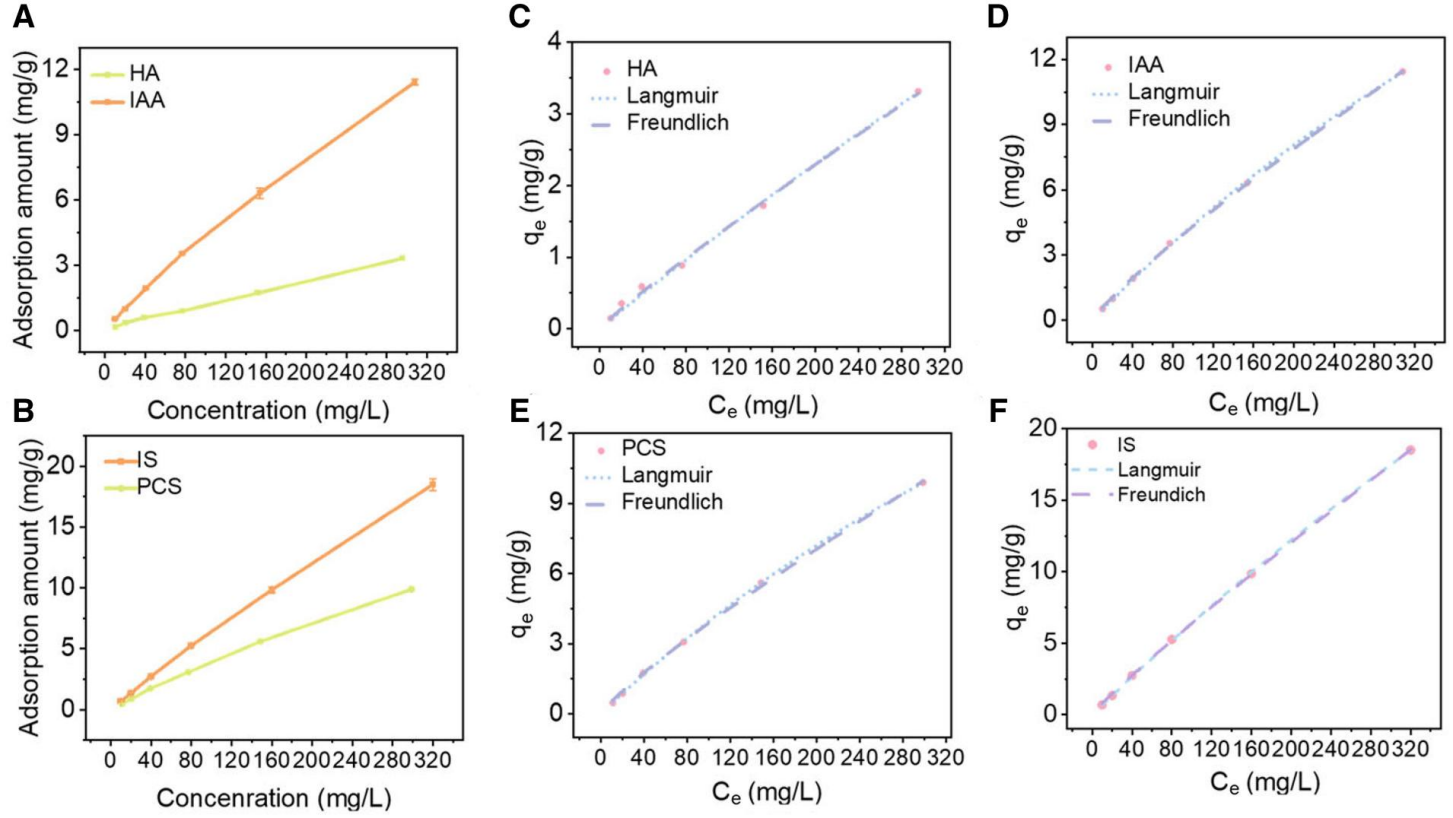
Adsorption isotherms of PBUTs by H-PES/SPSf@PEI microspheres. HA (**A**), IAA (**A**), PCS (**B**) and IS (**B**) adsorption amount of H-PES/SPSf@PEI microspheres at different initial concentrations (*n* = 3). Adsorption isotherms of (**C**) HA, (**D**) IAA, (**E**) PCS and (**F**) IS on H-PES/SPSF@PEI at a predetermined concentration. Freundlich equation and Langmuir parameters were fitted for adsorption equilibrium isotherms.

We next elucidated the adsorption isotherms of H-PES/SPSf@PEI microspheres for PBUTs. As shown in Figure 6A and B, the adsorption amount of PBUTs by H-PES/SPSf@PEI microspheres significantly increased with the increasing PBUTs concentration in a concentration-dependent manner, which could be attributed to an enhanced driving force of the concentration gradient. The adsorption isotherms were then fitted to both the Freundlich and Langmuir equations (Figure 6C–F), respectively, with corresponding adsorption isotherms parameters presented in Table 3. It can be observed that all PBUTs displayed a high coefficient of *R*^2^>0.95 for both Langmuir model and Freundlich model. The *K*_f_ values for the Freundlich model of H-PES/SPSf@PEI microspheres were as follows: HA<PCS<IAA<IS, suggesting that the affinity of H-PES/SPSf@PEI for PBUTs was in the order: HA< PCS<IAA <IS. The fit of adsorption to the Langmuir model indicating that the adsorption process was consistent with monolayer adsorption attributed to electrostatic interactions between PBUTs and H-PES/SPSf@PEI microspheres. The saturated adsorption amount (*Q*_max_) was also shown in Table 3, where *Q*_max_ of HA, PCS, IAA and IS was 34.24 mg/g, 40.31 mg/g, 49.19 mg/g and 128.67 mg/g, respectively. In summary, our results showed that H-PES/SPSf@PEI microspheres had good adsorption amount for major PBUTs.

**Table 3.** PBUTs Adsorption isotherms parameters of H-PES/SPSf@PEI microspheres

PBUTs	Freundlich			Langmuir		
	*R* ^2^	*K*_f_	*n* ^−1^	*R* ^2^	*Q*_max_ (mg/g)	b (L/mg)
HA	0.9973	0.0170	0.9257	0.9960	34.24	0.0004
IAA	0.9997	0.0802	0.8660	0.9996	49.19	0.0010
PCS	0.9992	0.0732	0.8619	0.9999	40.31	0.0011
IS	0.9999	0.0927	0.9184	0.9999	128.67	0.0005

**Figure 7.**
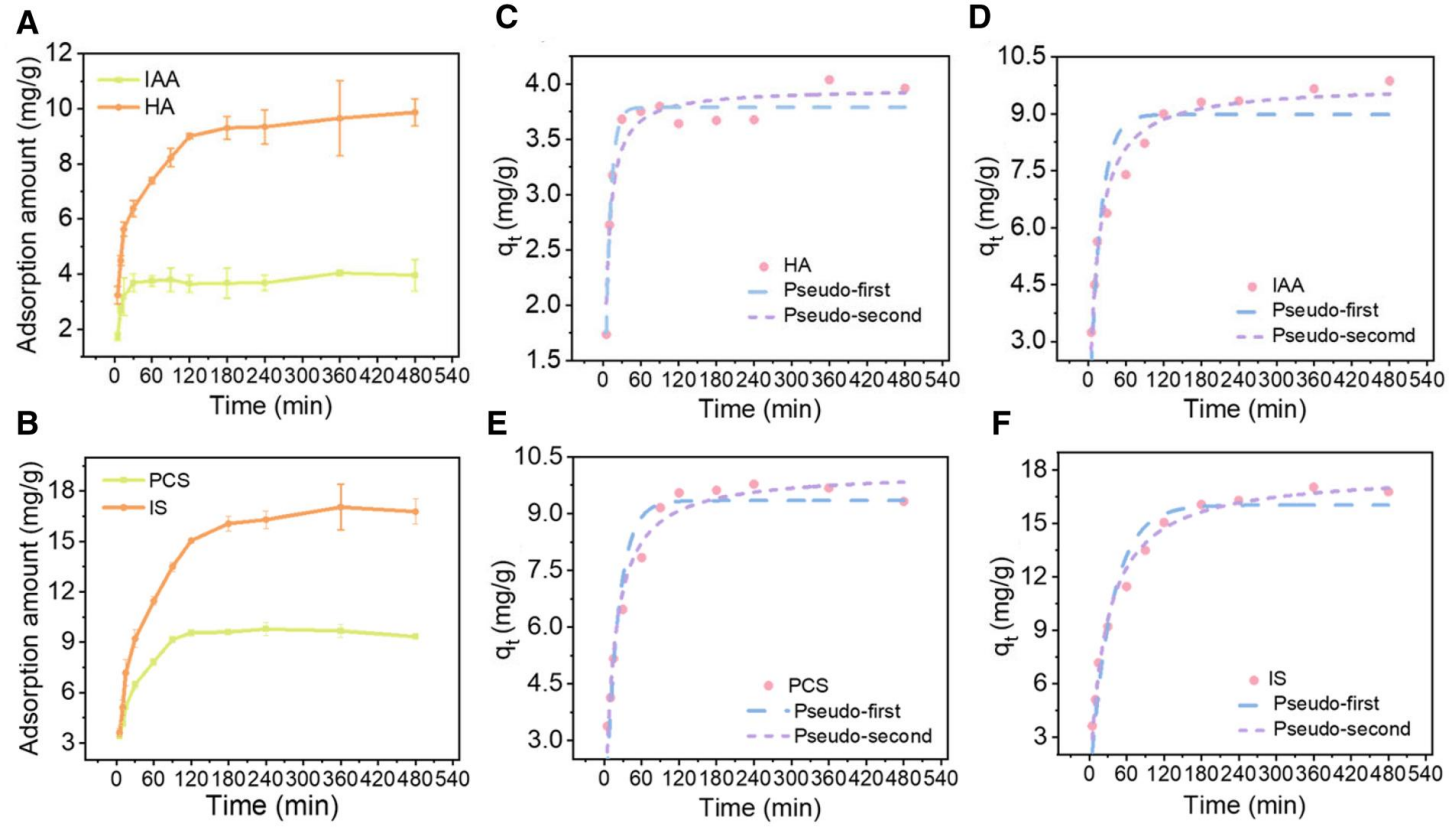
Adsorption kinetics of PBUTs by H-PES/SPSf@PEI microspheres. HA (**A**), IAA (**A**), PCS (**B**) and IS (**B**) adsorption amount of H-PES/SPSf@PEI microspheres at different time (*n* = 3). Adsorption kinetic curves of (**C**) HA, (**D**) IAA, (**E**) PCS and (**F**) IS, on H-PES/SPSF@PEI at 300 mg/L. Pseudo-first, and pseudo-second models were fitted for the curves.

Adsorption kinetics experiments were conducted to investigate the effect of adsorption time on the adsorption amount of 300 mg/L PBUTs by H-PES/SPSf@PEI microspheres. The adsorption amount of these PBUTs at different time points was fitted for pseudo-first and pseudo-second kinetic models. As shown in Figure 7A and B, the adsorption amount of four PBUTs by H-PES/SPSf@PEI reached equilibrium in ∼2 h, which was in line with a recommended treatment duration of hemoperfusion in ESRD patients [61]. The kinetic parameters are presented in Table 4. Among the two kinetic models, the pseudo-second model, with a larger regression coefficient *R*^2^>0.95, was better fitted for IAA, PCS and IS (Figure 7D–F), suggesting a classical chemical interaction process. However, as shown in Figure 7C, the pseudo-first model was better fitted for HA (*R*^2^: 0.9687 vs 0.9303), suggesting that HA adsorption was a physical adsorption process.

**Table 4.** PBUTs Adsorption kinetic model parameters of H-PES/SPSf@PEI

PBUTs	Pseudo-first kinetic model			Pseudo-second kinetic model		
	*R* ^2^	*q*_e_ (mg/g)	*k*_1_ (1/h)	*R* ^2^	*q*_e_ (mg/g)	*k*_2_ (g/(mg.h))
HA	0.9687	3.789	0.1236	0.9303	3.960	0.0525
IAA	0.8814	8.983	0.0594	0.9729	9.772	0.0082
PCS	0.9304	9.345	0.0509	0.9731	10.112	0.0072
IS	0.9490	16.042	0.0287	0.9886	17.915	0.0022

### Dynamic adsorption of PBUTs by H-PES/SPSf@PEI microspheres in a simulated hemoperfusion setting

**Figure 8.**
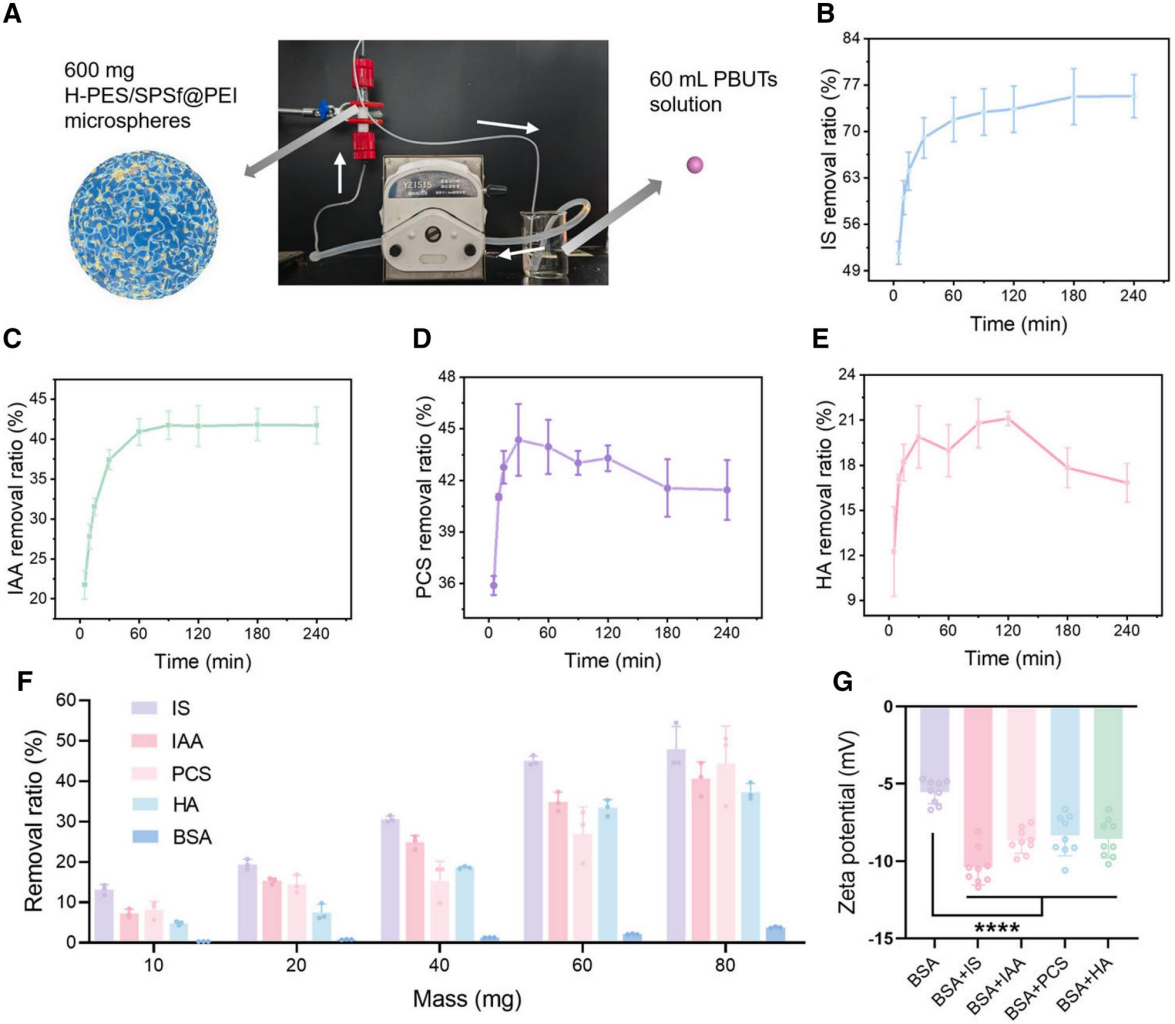
Simulated hemoperfusion and competitive adsorption of PBUTs by H-PES/SPSf@PEI microspheres. (**A**) The schematic diagram of dynamic perfusion device. Removal ratio (IS (**B**), IAA (**C**), PCS (**D**) and HA (**E**)) of the perfusion cartridge loaded with H-PES/SPSf@PEI in 25 mg/L PBUTs solution (dissolved in PBS) (*n* = 3). (**F**) Competitive adsorption study by H-PES/SPSf@PEI of different masses in mixed PBUTs and BSA solution (*n* = 3). (**G**) Zeta potentials of BSA solutions and BSA-bound PBUTs solutions (removing free PBUTs) in PBS (*n* = 9). **** For *P *< 0.0001.

To verify the application potential of H-PES/SPSf@PEI microspheres in clinical practice for the removal of PBUTs, a simulated hemoperfusion session was conducted. The hemoperfusion simulation setup is illustrated in Figure 8A. PBUTs solution was pumped through a home-made hemoperfusion cartridge containing 600 mg of H-PES/SPSf@PEI microspheres for 4 h. Concentrations of PBUTs solution in this study were set according to the serum levels of major PBUTs observed in CKD patients [37, 38] (The concentration of IAA was slightly higher than the levels in CKD patients).

As shown in Figure 8B and C, the removal ratio of IS and IAA after 1-h simulated hemoperfusion was 71.80% and 40.92%, which increased slightly to 75.33% and 41.73% after 3 h. In contrast, the simulated hemoperfusion cartridge achieved saturation adsorption of PCS within 30 min, reaching a maximum removal efficiency of 44.36% (Figure 8D). Subsequently, the PCS removal ratio exhibited a gradual decline, paralleled by a similar trend observed for HA adsorption (Figure 8E), suggesting potential desorption dynamics at later time points. This may be due to an insufficient affinity of H-PES/SPSf@PEI microspheres to PCS and HA, leading to the leak of adsorbed PCS and HA from H-PES/SPSf@PEI microspheres at a high flow rate. These results indicated that the simulated hemoperfusion cartridge equipped with H-PES/SPSf@PEI microspheres had a favorable removal ratio for PBUTs in a dynamic simulated hemoperfusion setting.

### Competitive adsorption of PBUTs by H-PES/SPSf@PEI microspheres in BSA solution

To evaluate the adsorption amount of PBUTs by H-PES/SPSf@PEI microspheres in a more realistic biological environment, competitive adsorption study was conducted. In this study, BSA was used as a substitute for albumin to investigate the competitive adsorption performance by H-PES/SPSf@PEI microspheres. As shown in Figure 8F, the removal ratio of PBUTs increased with the mass of H-PES/SPSf@PEI microspheres in a dose-dependent manner. To be specific, 80 mg of H-PES/SPSf@PEI microspheres could remove 47.89% IS, 40.64% IAA, 44.42% PCS and 37.35% HA in BSA solution. In contrast, only 3.80% of BSA was tightly bound by the H-PES/SPSf@PEI microspheres. This may be due to the fact that BSA bound to PBUTs has a more negative Zeta potential (Figure 8G). Therefore, we hypothesize that, in a mixed solution of PBUTs and BSA, H-PES/SPSf@PEI microspheres preferentially tend to adsorb free PBUTs and BSA-bound PBUTs. When free PBUTs are adsorbed, the dissociation of PBUTs and BSA will be promoted, allowing H-PES/SPSf@PEI microspheres prefer to adsorb PBUTs rather than BSA. In conclusion, our data showed that H-PES/SPSf@PEI microspheres preferentially adsorb PBUTs in a protein solution with a high affinity.

### A state-of-the-art comparison of currently available hemoperfusion adsorbents for the removal of PBUTs

The development of effective hemoperfusion adsorbents for the removal of PBUTs is a key to improving the long-term prognosis of ESRD patients. As shown in Supplementary Table S2, although multiple adsorbents derived from activated carbon, zeolites and MOFs have been developed, they often have obvious disadvantages, such as complex preparation processes, poor hemocompatibility and limited adsorption specificity in protein solutions. Compared with recent studies, H-PES/SPSf@PEI microspheres exhibited favorable hemocompatibility and satisfying adsorption amount for major PBUTs. Most importantly, compared to BSA, H-PES/SPSf@PEI microspheres effectively removed PBUTs in a high-concentration protein solution, making it possible to use H-PES/SPSf@PEI microspheres as a promising treatment option for ESRD patients with elevated plasma concentrations of PBUTs. Our future work will focus on the optimization of the surface chemistry and structure of H-PES/SPSf@PEI microspheres to enhance their affinity for PBUTs and to prevent non-specific protein adsorption in plasma.

## Conclusion

In this work, we developed adsorptive H-PES/SPSf@PEI microspheres by blending PES, SPSf and PEI to efficiently eliminate PBUTs via electrostatic interactions. The addition of sulfuric acid in the coagulation bath was crucial to regulate the surface charge and structure of the microsphere, leading to smaller pore sizes and neutralization of the surface charge. As tailored hemoperfusion adsorbents for PBUTs, our H-PES/SPSf@PEI microspheres had favorable hemocompatibility, as evidenced by insignificant hemolysis, activation of the complement system and platelets. Through adsorption isotherm studies, we showed that the maximum adsorption amounts of HA, IAA, PCS and IS by H-PES/SPSf@PEI microspheres were 34.24 mg/g, 49.19 mg/g, 40.31 mg/g, 128.67 mg/g, respectively. Moreover, H-PES/SPSf@PEI microspheres could also effectively remove PBUTs both in a simulated hemoperfusion setting and in BSA solution, indicating the superior affinity between PBUTs and H-PES/SPSf@PEI microspheres. In conclusion, H-PES/SPSf@PEI microspheres hold a potential to eliminate PBUTs from patients with ESRD.

## Supplementary Material

rbaf082_Supplementary_Data
