# Supplementary material for: A highly biocompatible polyethyleneimine/sulfonated polysulfone hemoperfusion microsphere with tailored surface charge for rapid and efficient removal of major protein-bound uremic toxins from simulated human plasma
Source: Regen Biomater. 2025 Aug 8;12:rbaf082. doi: 10.1093/rb/rbaf082 (PMC12582388; doi:10.1093/rb/rbaf082)
Supplement: rbaf082_Supplementary_Data [file rbaf082_supplementary_data.docx]

**Supplementary data for**

**A highly biocompatible polyethyleneimine/sulfonated polysulfone hemoperfusion microsphere with tailored surface charge for rapid and efficient removal of major protein-bound uremic toxins from simulated human plasma**

Shujing Wang ^a^, Jiahao Liang ^b^, Yu Chen ^b^, Xianda Liu ^b^, Dongmei Tong ^b^, Yupei Li ^c,*^, Weifeng Zhao ^b^, Baihai Su ^a,c,d,e,*^, Changsheng Zhao^b^

^a^ Department of Nephrology, Kidney Research Institute, Frontiers Science Center for Disease-related Molecular Network, West China Hospital of Sichuan University, Chengdu, 610041, China

^b^ College of Polymer Science and Engineering, State Key Laboratory of Advanced Polymer Materials (Sichuan University), Chengdu, 610065, China

^c^ Department of Nephrology, Kidney Research Institute, West China Hospital of Sichuan University, Chengdu 610041, China

^d^ Med+ Biomaterial Institute of West China Hospital/West China School of Medicine, Sichuan University, Chengdu 610041, China.

^e^ Med-X Center for Materials, Sichuan University, Chengdu 610041, China.

*Corresponding author.

1. **Experiment section**
   1. **Characterization of H-PES/SPSf@PEI microspheres**
      1. **Scanning electron microscopy (SEM)**

The microspheres were frozen at -40°C for 12 h and then freeze-dried. The cross-section samples were quick-frozen with liquid nitrogen and then cut with a blade. The lyophilized samples were sprayed with gold on the surfaces by ion sputtering (vacuum degree 8 kPa, current 6-8 mA), and then observed by a scanning electron microscope (Regulus 8220, Japan).

- - 1. **Fourier transform infrared spectroscopy (FTIR)**

The microspheres were dried in an oven at 60°C for 18 h to remove moisture. The pressed sample tablets were detected using the reflective mode of an FTIR spectrometer (Thermo Fisher Nicolet Is50, USA) with a scanning range of 4000 500 cm^−1^ and a resolution of 2 cm^−1^.

- - 1. **X-ray photoelectron spectroscopy (XPS)**

The microspheres were freeze-dried and detected by an X-ray photoelectron spectrometer (XPS, Thermo Fisher K-ALPHA, USA). The excitation source was Al kα ray (hν = 1486.6 eV). Thermo Scientific™ Avantage was used to analyze the resulting data.

- - 1. **Thermogravimetric analysis (TGA)**

The microspheres were freeze-dried. Then, 5~10 mg of dried microspheres was weighed and added into a ceramic crucible. The TGA curves were obtained by a thermogravimetric analyzer (TGA, METTLER TOLEDO, Switzerland). The heating program was from 30°C to 800°C at a heating rate of 10 °C/min. The entire test was carried out under N_2_ atmosphere.

- - 1. **Mercury intrusion porosimetry (MIP)**

Mercury intrusion porosimetry (MIP, AutoPore IV 9500, USA) was employed to determine the porosity and pore distribution. Lyophilized microspheres were used for testing, The pore diameter was calculated by the Washburn equation (**Equation S1**)

D = -$\frac{4\gamma cos \theta}{P}$ (S1)

where D is the pore diameter, γ is the surface tension of mercury (484 dyn/cm), θ is the contact angle of mercury on the surface of the sample (130° in the experiment), P is the applied pressure.

To determine the pore size distribution of the hydrogel spheres, the critical pressure Pc is defined as the minimum pressure required to intrude the largest hole2 which is determined by **Equation S2**

ln[V(∞)-V(p)] = ln[V(∞) - V(p)] - m[ln(P) - ln(Pc)] (S2)

where V(∞) is the volume of mercury immersed at maximum pressure, V(p) is the volume of mercury immersed at P, m is the slope obtained from the equation.

- - 1. **Compressive strength**

The compressive strength was tested using a universal testing machine (HZ-1003-DZ, China) equipped with a 10-kg sensor.

**1.2 Hemocompatibility experiments**

**1.2.1 Bovine serum albumin adsorption**

50 mg of different microspheres were immersed in PBS buffer for 12 h before the experiment, then PBS buffer was removed. 1 mL of 1 mg/mL bovine serum albumin (BSA) solution was added to incubate with pre-treated microspheres at 37°C for 1 h. Subsequently, the microspheres were rinsed with PBS buffer and DI water three times, respectively, to remove unbound BSA. Then, the microspheres were placed in 2 mL of 2 wt.% sodium dodecyl sulfate solution at 37℃ for another 2 h with vigorous oscillation to remove adsorbed BSA. A BCA protein assay kit (Thermo Fisher Pierce) was used to measure the concentration of protein in sodium dodecyl sulfate solution. BSA adsorption amount was calculated using the following equation:

BSA adsorption amount (mg/g) = $\frac{C_{o}\times V}{M}$ (S3)

where C_o_ is the concentration of protein in the washing solution, V is the volume of sodium dodecyl sulfate solution, M is dry weight of the microspheres.

**1.2.2 Hemolysis ratio**

Fresh blood anticoagulated with sodium citrate was centrifuged at 2000 rpm for 10 min to obtain red blood cells (RBCs). The obtained RBCs were then washed with normal saline five times. The RBCs were diluted 20-fold with normal saline to prepare a diluted RBC suspension. 50 mg of microspheres were immersed in normal saline for 30 min, then the normal saline was removed, and 200 µL RBC suspension and 800 µL normal saline was added to incubate with the microspheres at 37°C for 3 h under shaking. Afterwards, the RBC suspension was centrifuged at 7000 rpm for 3 min, then the supernatant was collected to measure the absorbance at 540 nm. 800 µL of DI water and normal saline were added to 200 µL of RBC suspension to prepare positive and negative control, respectively. The hemolysis ratio was described as the following equation:

Hemolysis ratio (%) = $\frac{A_{s}-A_{n}}{A_{p}-A_{n}}\times100\%$ (S4)

where A_s_ is the absorbance of sample, A_p_ and A_n_ represent the absorbance of positive sample and negative controls at 540 nm.

**1.2.3 Blood routine test**

10 mg of different microspheres were immersed in PBS buffer for 12 h before the experiment. Then, PBS buffer was removed and the microspheres were incubated with 200 µL of EDTA-anticoagulated fresh blood at 37℃ for 1 h. Afterwards, microsphere-treated blood samples were analyzed by an automated hematology analyzer (Mindray BC-5100, China).

**1.2.4 Platelet adhesion determined by the LDH assay**

Fresh blood anticoagulated with sodium citrate was centrifuged at 800 rpm for 10 min to prepare platelet-rich plasma (PRP). 15 mg of different microspheres were pre-immersed in PBS buffer for 12h, then PBS buffer was removed. Afterwards, the pre-treated microspheres were incubated with 150µL of PRP for 1 h. Afterwards, the acquired plasma was tested by the corresponding LDH assay kit.

**1.2.5 Platelet morphology**

15 mg of different microspheres were immersed in PBS buffer for 12 h before PBS buffer was removed. The microspheres were then incubated with 150µL of PRP for 2h. After incubation, the microspheres were fixed with paraformaldehyde, dehydrated through gradients. Finally, the morphology and number of adhered platelets on the surface of the microspheres were observed by SEM.

**1.2.6 Complement activation**

Plasma concentrations of C3a and C5a were measured by corresponding enzyme-linked immune sorbent assay (ELISA) kits (Thermo Fisher Human C3a ELISA Kit and CUSABIO Human C5a ELISA Kit). 7.5 mg of different microspheres were immersed in PBS buffer for 12 h before the experiment. Then the pre-treated microspheres were incubated with 150 µL of blood (anticoagulated with sodium citrate and recombinant hirudin to ensure the progress of complement activation pathway and to avoid blood clotting) at 37℃ for 1 h. The blood incubated with cobra venom factor was served as a positive control. Then, the blood was centrifuged at 7000 rpm for 10 min to obtain the testing plasma. Finally, the acquired plasma was diluted and tested by corresponding ELISA kits according to the instruction manual.

**1.2.7 Clotting times**

Fresh blood anticoagulated with sodium citrate was centrifuged at 4000 rpm for 15 min to prepare platelet-poor plasma (PPP). 30 mg of different microspheres were immersed in PBS buffer for 12h before the experiment. Then, PBS buffer was removed, and the microspheres were incubated with 300 μL of PPP at 37°C for 30 min. Afterwards, activated partial thromboplastin time (APTT), thrombin time (TT), prothrombin time (PT) and the concentration of fibrinogen of PPP were measured by an automatic blood coagulation analyzer (CA500, Sysmex, Japan), the corresponding test regents were purchased from SIEMENS Co., Ltd.


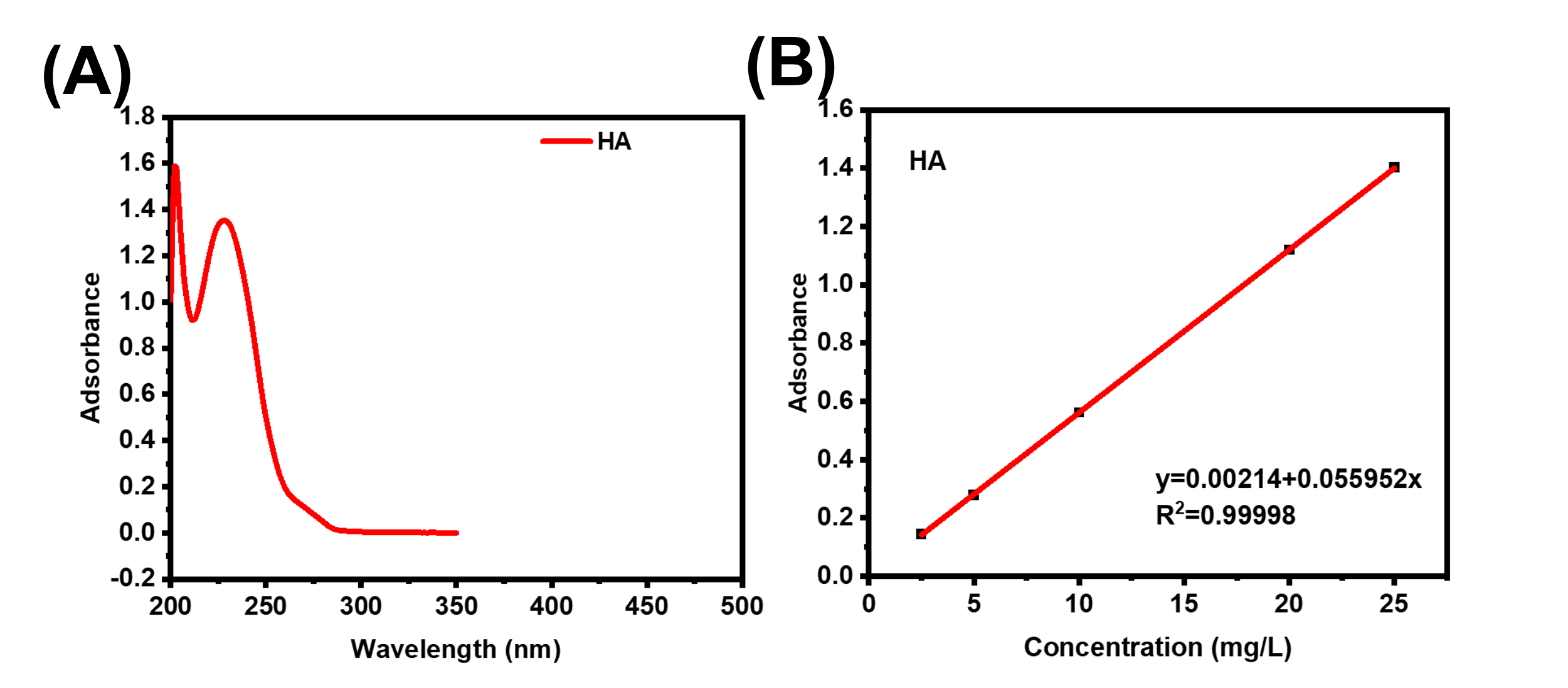


**Supplementary Figure 1.** (A) UV spectra of HA. (B) Standard curves of HA.


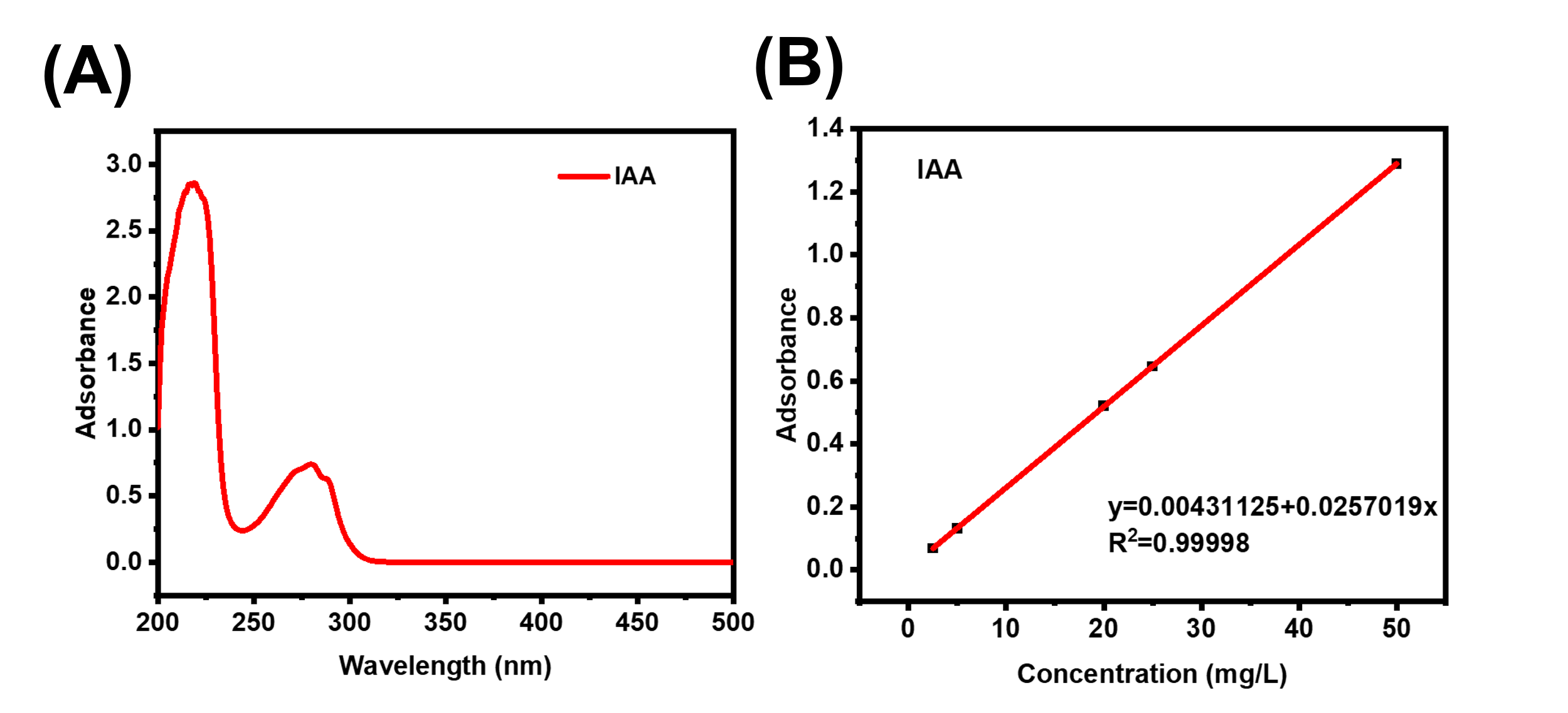


**Supplementary Figure 2.** (A) UV spectra of IAA. (B) Standard curves of IAA.


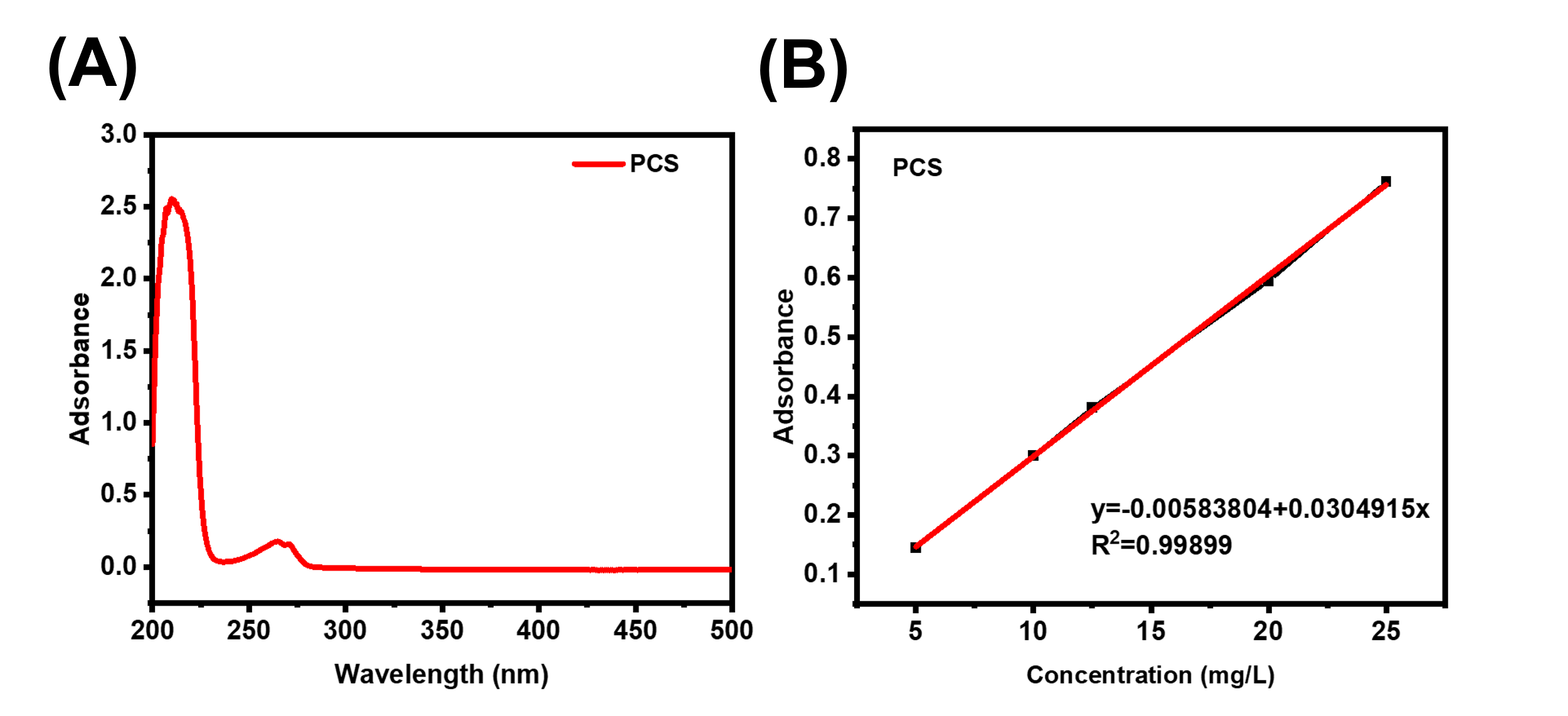


**Supplementary Figure 3.** (A) UV spectra of PCS. (B) Standard curves of PCS.


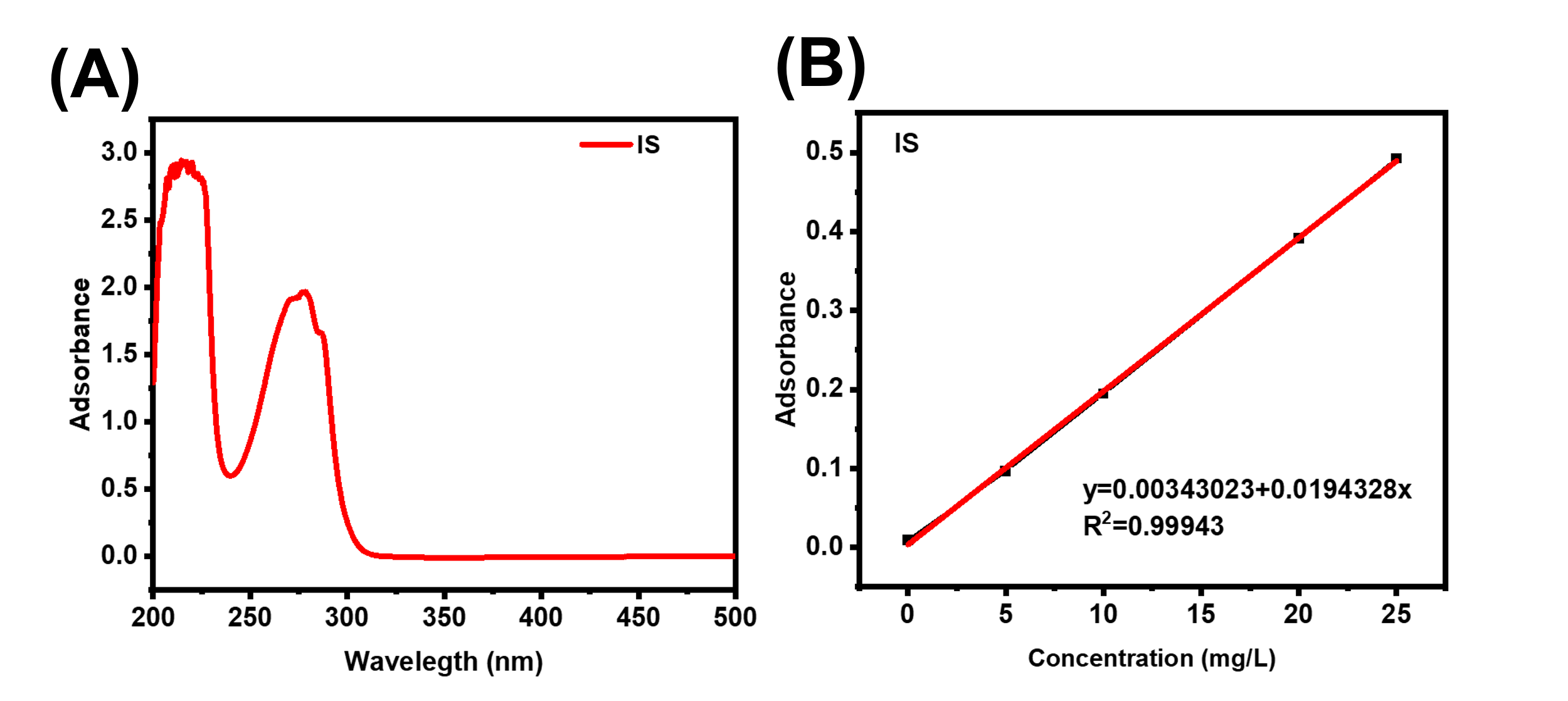


**Supplementary Figure 4.** (A) UV spectra of IS. (B) Standard curves of IS.


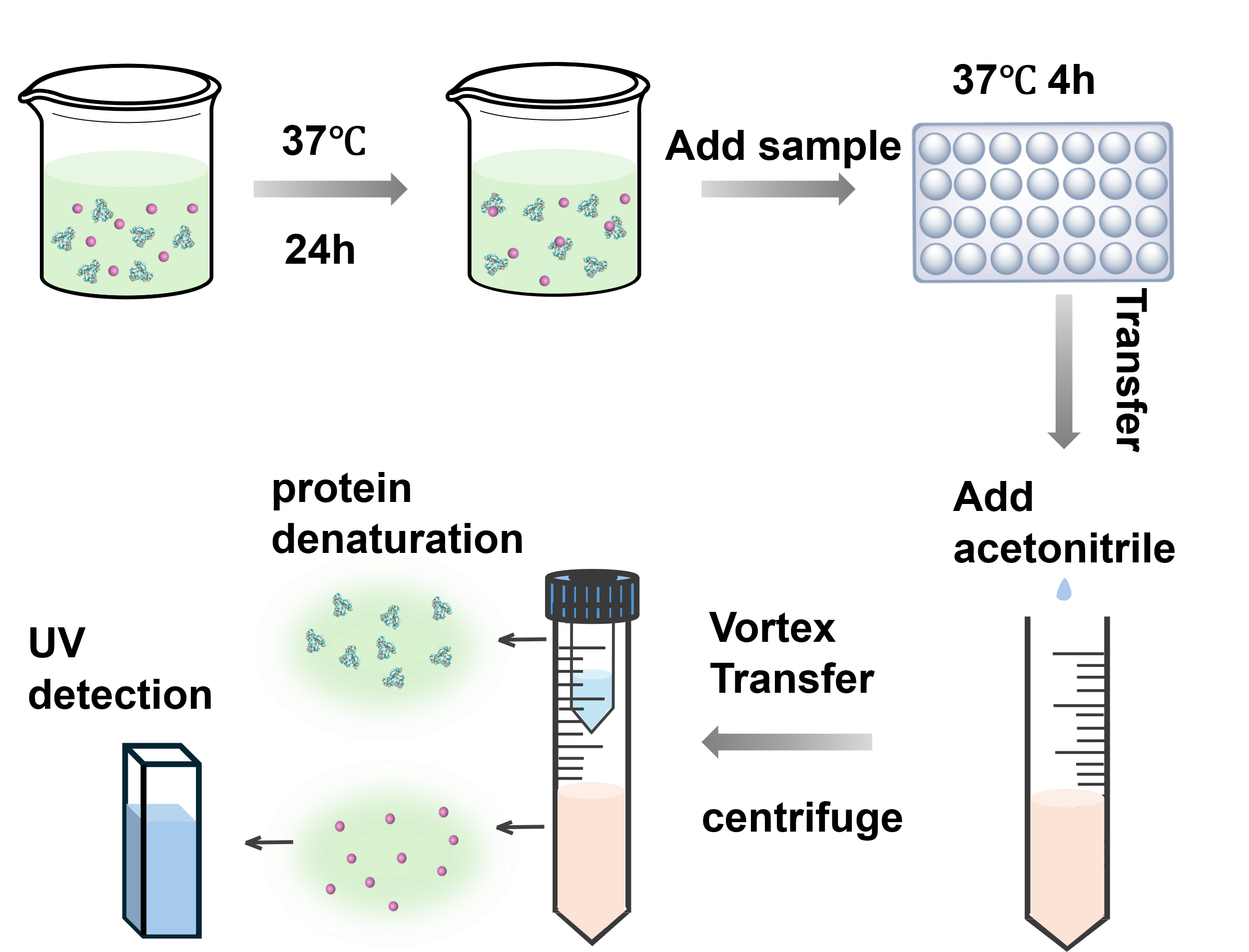


**Supplementary Figure 5.** Schematic illustration of competitive adsorption and detection of PBUTs concentration in BSA solution.

1. **Results and discussions**

**Supplementary Table 1.** Porosity, total pore area at 32993.31 psia, and average pore size of all microspheres.

| Sample | Porosity (%) | Total pore area at 32993.31 psia (m^2^/g) | Average pore size (4V/A, nm) |
| --- | --- | --- | --- |
| H-PES | 79.6696 | 103.655 | 107.80 |
| H-PES/SPSf | 60.1728 | 80.221 | 54.40 |
| H-PES/PEI | 81.3171 | 82.859 | 172.76 |
| PES/SPSF@PEI | 83.5782 | 82.986 | 225.69 |
| H-PES/SPSF@PEI | 77.1944 | 124.799 | 80.45 |


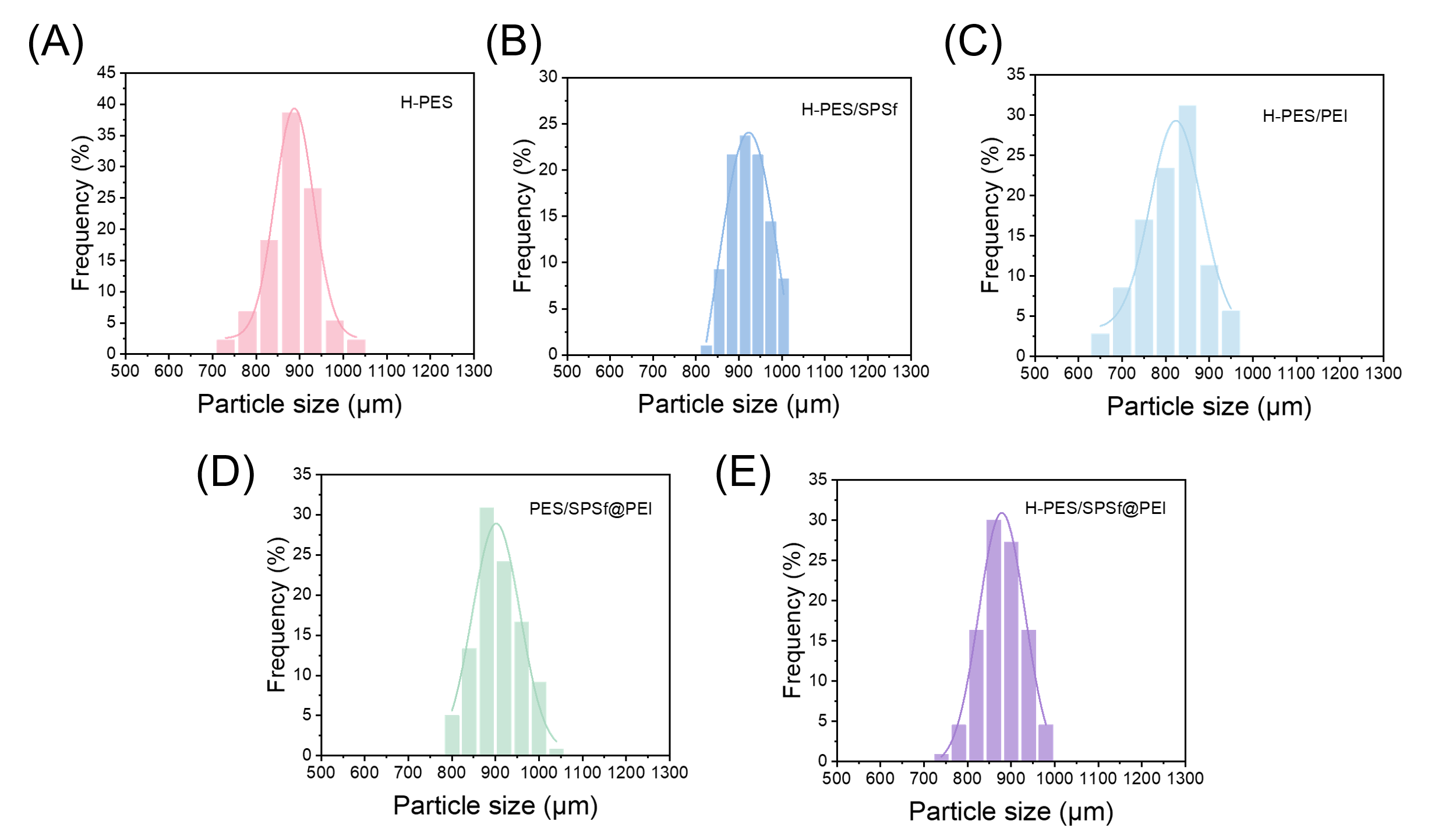


**Supplementary Figure 6.** Particle size distribution and probability density function fitting curves of (A) H-PES, (B) H-PES/SPSf, (C) H-PES/PEI, (D) PES/SPSf@PEI and (E) H-PES/SPSf@PEI microspheres.


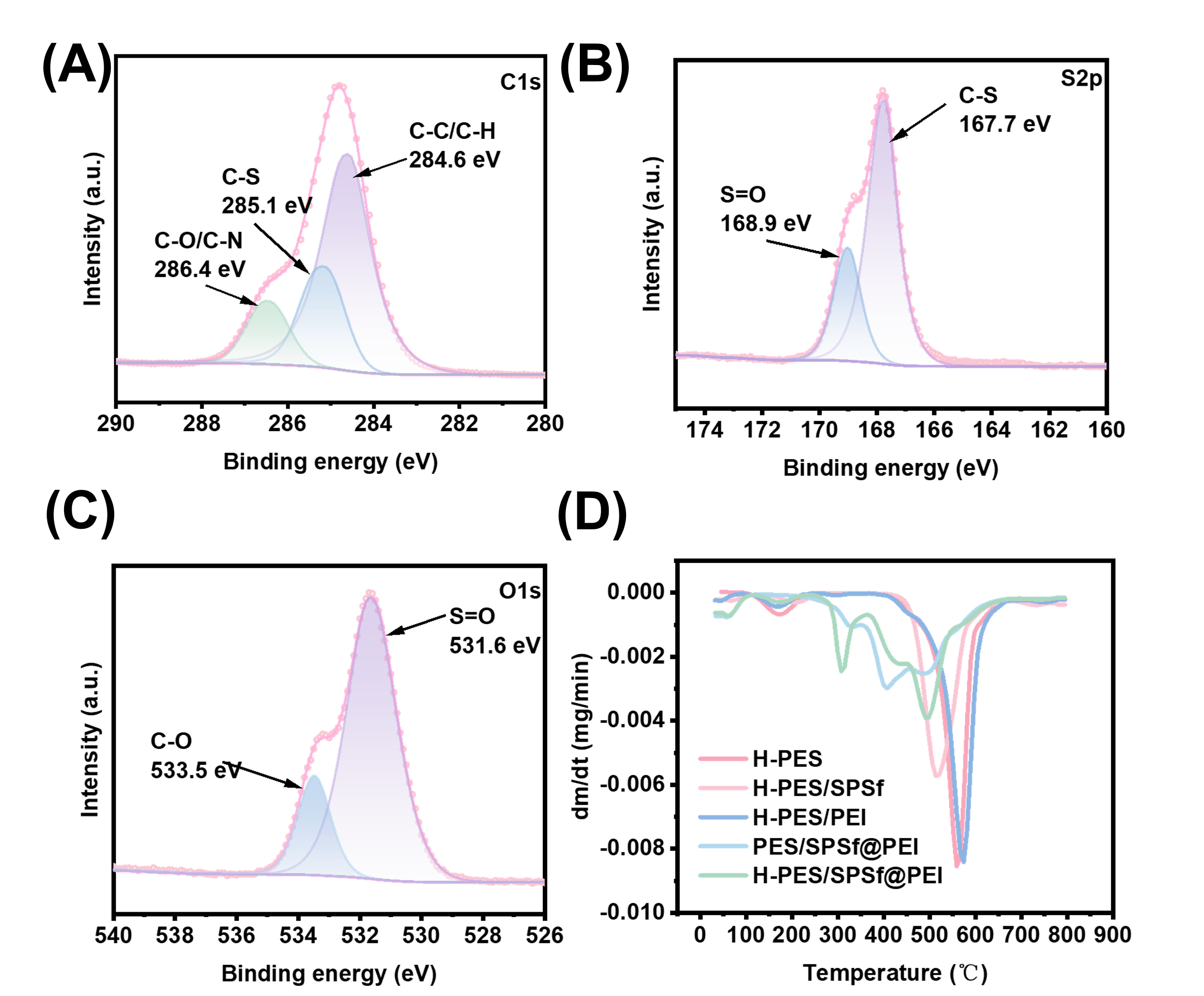


**Supplementary Figure 7.** The high-resolution XPS spectra of C1s (A), S2p (B), and O1s (C) of H-PES/SPSf@PEI microspheres. (D) DTG curves of H-PES, H-PES/SPSf, H-PES/PEI, PES/SPSf@PEI and H-PES/SPSf@PEI microspheres.


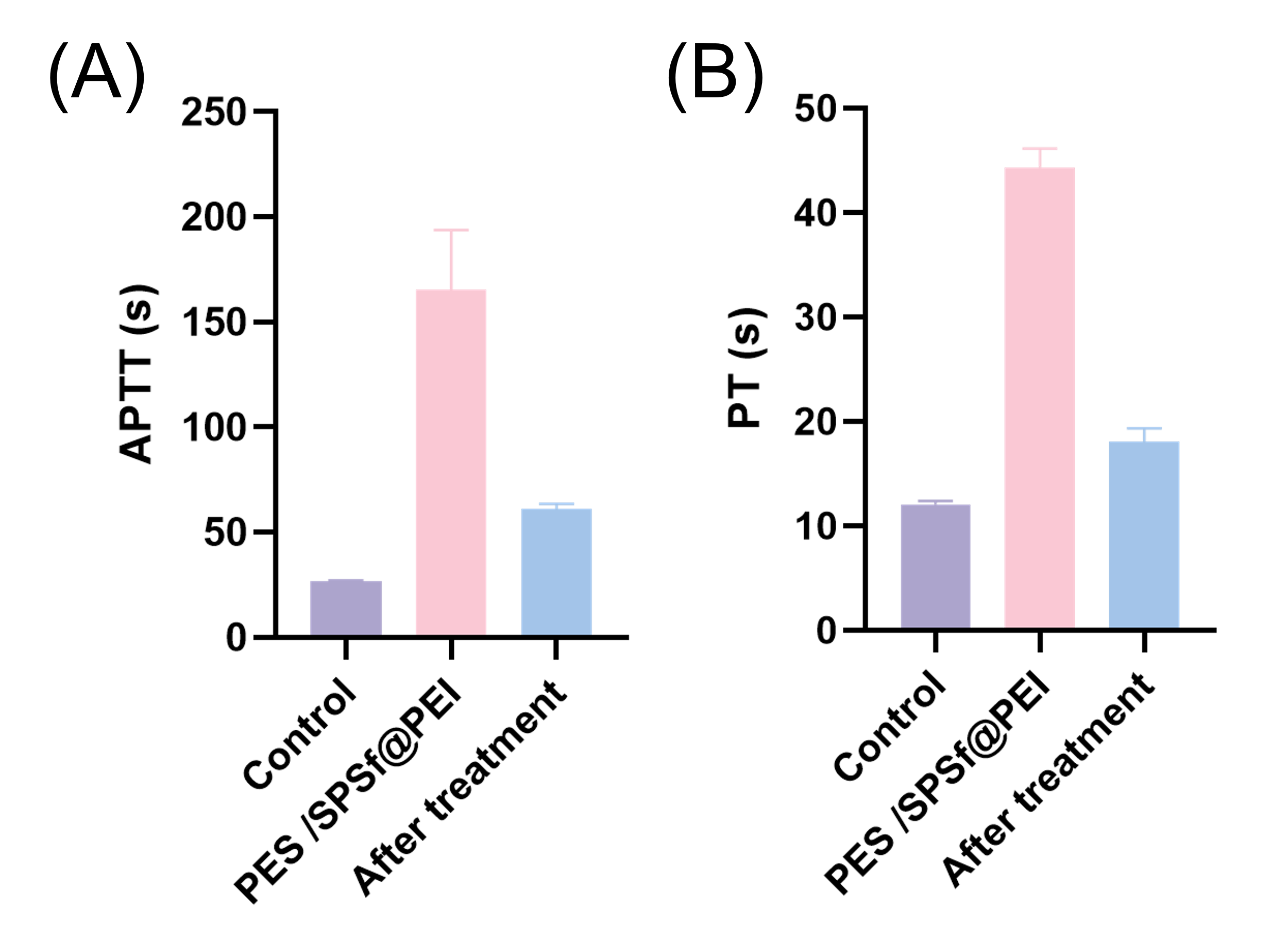


**Supplementary Figure 8.** APTT (A) and PT (B) values of PES/SPSf@PEI microspheres before and after sulfuric acid treatment (n=3).


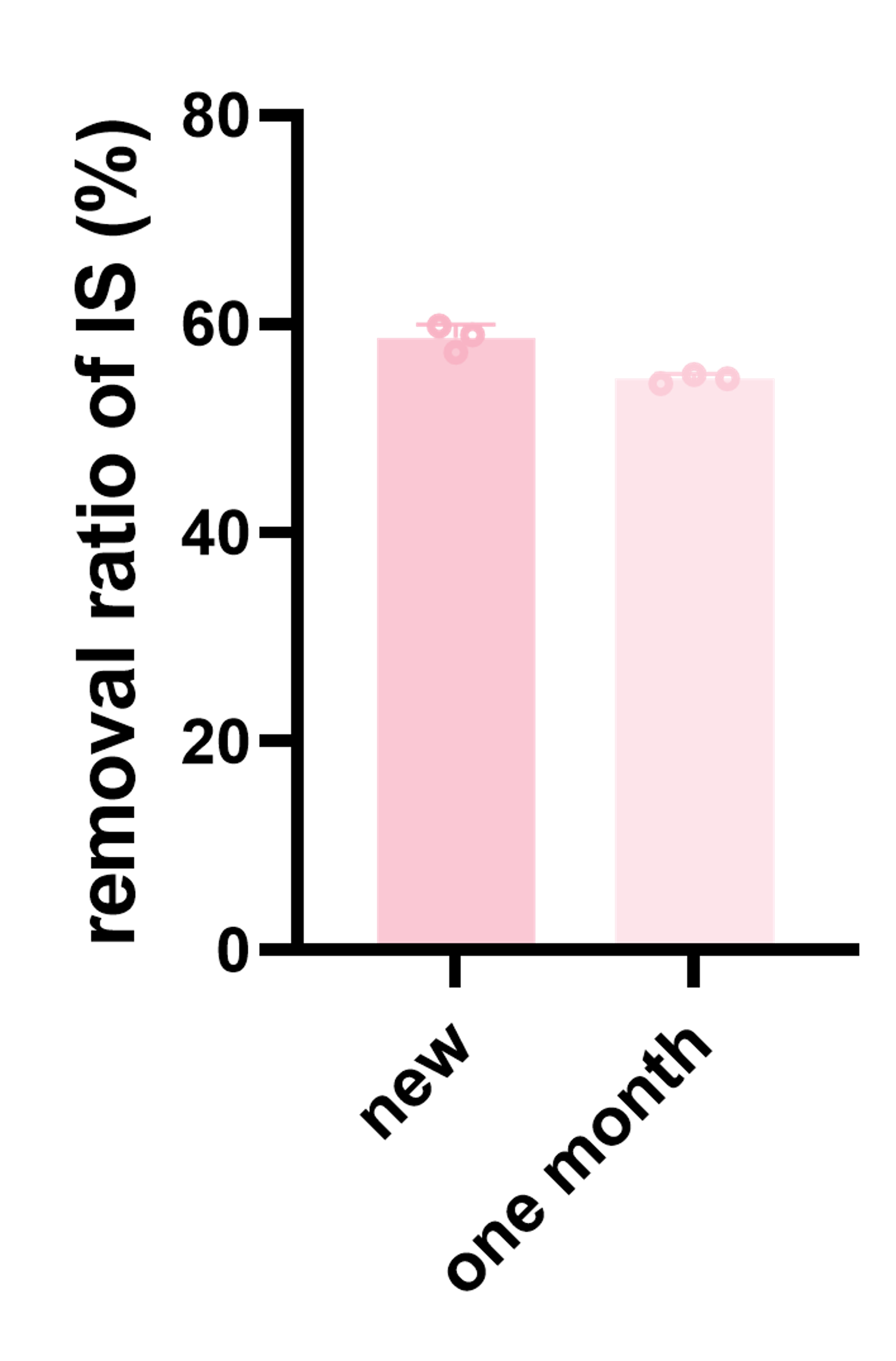


**Supplementary Figure 9**. Removal ratio of IS by H-PES/SPSf@PEI microspheres before and after one month (n=3).


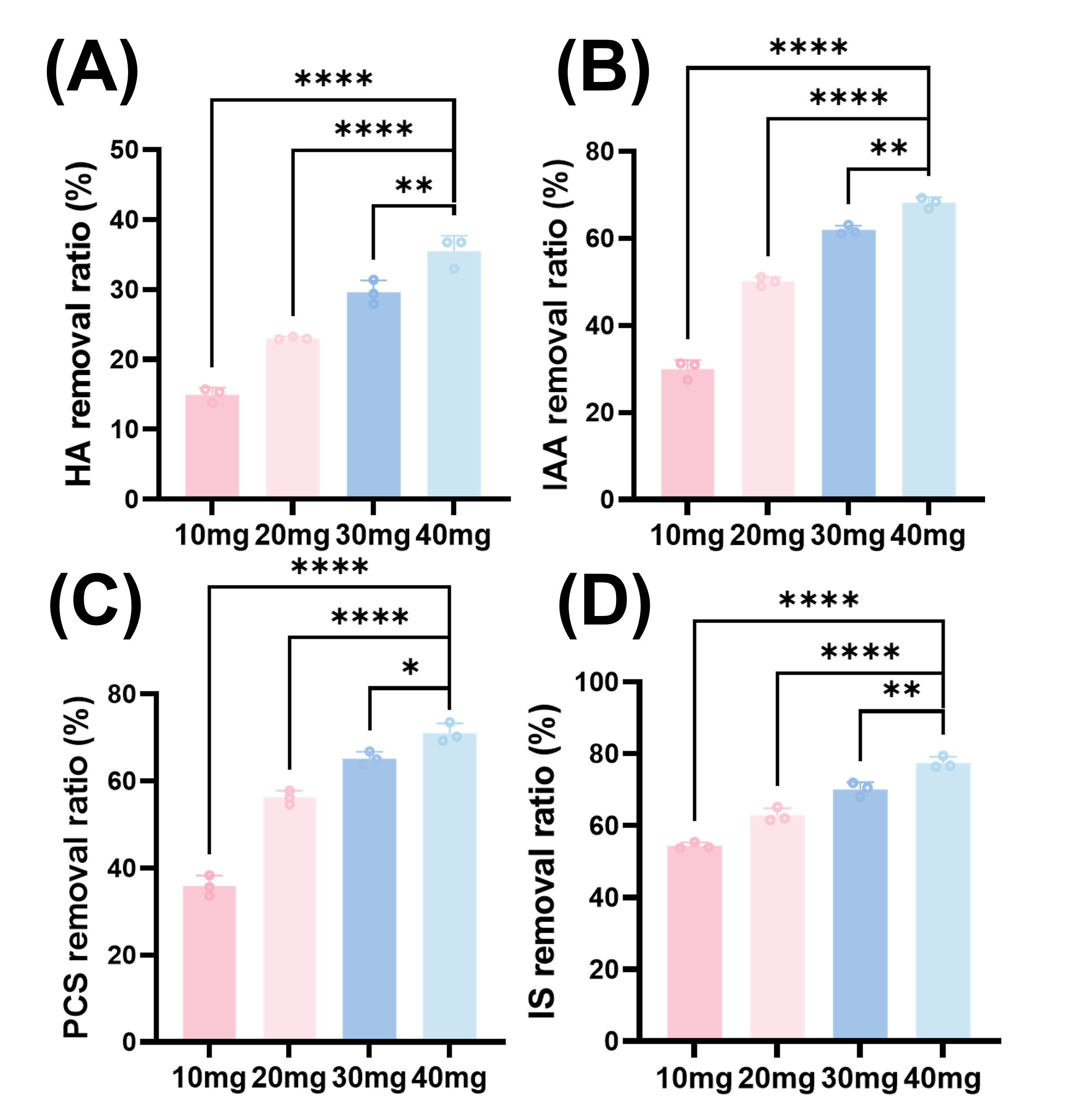


**Supplementary Figure 10.** Removal ratio of HA (A), IAA (B), PCs (C) and IS (D) by H-PES/SPSf@PEI of different mass (n=3).

**Supplementary Table 2.** A state-of-the-art comparison of currently available hemoperfusion adsorbents for the removal of PBUTs

| **References** | **Adsorbent modality** | **PBUTs adsorption capacity** | **Adsorption in protein solution** | **Hemolysis** | **Blood cell counts** | **Protein adsorption** | **Platelet adhesion** | **Complement activation** | **Clotting times** |
| --- | --- | --- | --- | --- | --- | --- | --- | --- | --- |
| **This work** | microsphere | HA (39.4 mg/g)  IAA (63.1 mg/g)  PCs (33.0 mg/g)  IS (128.67 mg/g)  In PBS | √ | √ | √ | √ | √ | √ | √ |
| **[1]** | aerogel | IS (69.4 mg/g) | √ | × | × | × | × | × | × |
| **[2]** | flat membrane | HA (78 mg/g)  PCs (134mg/g)  IS (183 mg/g)  In water | √ | × | × | × | SEM observation | × | √ |
| **[3]** | nanoparticle | HA (38.3 mg/g)  IS (49.5 mg/g)  In Tyrode buffer | × | √ | × | × | SEM observation | × | × |
| **[4]** | liposome | IS (24.15 mg/g)  In PBS | √ | √ | × | × | × | × | × |
| **[5]** | particle | PCS (1000.8 mg/g)  IS (1028.4 mg/g)  in PBS | √ | √ | × | × | × | × | √ |
| **[6]** | particle | HA （129.8 mg/g）  IS （63.1 mg/g）  in water | × | × | × | × | × | × | × |
| **[7]** | nanoparticle | PCS （282 mg/g）  IS （329 mg/g）  Qa （188 mg/g）  In water | √ | √ | × | √ | × | × | √ |
| **[8]** | nanoparticle | PCS (197.2 mg/g)  IS (230.4 mg/g)  In water | √ | × | × | × | × | × | × |
| **[9]** | microsphere | IS (166.89 mg/g)  PCS (84.1 mg/g)  IAA (208.93 mg/g)  In PBS | √ | √ | × | √ | × | × | √ |
| **[10]** | nanocage | IS (458 μg/g)  PCS (372 μg/g)  PheAc (2710μg/g) | × | × | × | × | × | × | × |

**References**

1. Jin YY, Ding SP, Li PY, Wang XF. Coordination of thin-film nanofibrous composite dialysis membrane and reduced graphene oxide aerogel adsorbents for elimination of indoxyl sulfate. *Chinese Journal of Chemical Engineering* 2022;49:111-121.

2. Liu Y, Li GL, Han Q, Lin HB, Deng G, Li Q, Liu F. Designing adsorptive membranes for removing protein-bound uremic toxins via π-π and cation-π interaction. *Journal of Membrane Science* 2023;676:12.

3. Zeng S, Hou YQ, Zhou YM, Zhou X, Ye SF, Wang M, Ren L. Adsorptive removal of uremic toxins using Zr-based MOFs for potential hemodialysis membranes. *Journal of Materials Science* 2022;57:2909-2923.

4. Shen YQ, Shen Y, Li JL, Ding F, Wang YF. Polyethyleneimine-anchored liposomes as scavengers for improving the efficiency of protein-bound uremic toxin clearance during dialysis. *Journal of Biomedical Materials Research Part A* 2022;110:976-983.

5. Zhang MY, Miao YJ, Zhang P, Xiao CL. Clearance of Protein-Bound Uremic Toxins Using Anion Nanotraps with Record High Uptake. *Acs Applied Materials & Interfaces* 2024;16: 68426-68436.

6. Ding SP, Wang D, Wang XF. Hierarchically structural layered double oxides with stretchable nanopores for highly effective removal of protein-bound uremic toxins. *Separation and Purification Technology* 2022;301:122033.

7. Chao ZH, Li JY, Jiang WN, Zhang C, Ji JX, Hua X, Xu L, Han LL, Jia LY. Hemocompatible MOF-decorated pollen hemoperfusion absorbents for rapid and highly efficient removal of protein-bound uremic toxins. *Materials Chemistry Frontiers* 2021;5:7617-7627.

8. Zhang MY, Li L, Lei LC, Kang K, Xiao CL. Effectively Decontaminating Protein-Bound Uremic Toxins in Human Serum Albumin Cationic Frameworks. *Acs Applied Materials & Interfaces* 2022;14:55354-55364.

9. Liu YH, Peng XY, Hu ZD, Yu MG, Fu JJ, Huang YG. Fabrication of a novel nitrogen-containing porous carbon adsorbent for protein-bound uremic toxins removal. *Materials Science & Engineering C-Materials for Biological Applications* 2021;121: 111879.

10. Böhler H, Orth-Alampour S, Baaten C, Riedner M, Jankowski J, Beck T. Assembly of chemically modified protein nanocages into 3D materials for the adsorption of uremic toxins. *Journal of Materials Chemistry B* 2022;11:55-60.
